# Dietary targeting of CNBP to rein in the EREG-EGFR cascade and restore homeostasis in colitis-associated colorectal cancer

**DOI:** 10.7150/ijbs.128354

**Published:** 2026-02-26

**Authors:** Tao Zhang, Lisha Zhou, Boyang Wang, Meijing Wang, Yuyu Zhu, Yitong Liu, Tingyu Zhang, Qingyuan Liu, Chuang Xiao, Lingdong Kong, Qiang Xu, Bo Zhang, Jiao Qu, Shao Li, Yang Sun

**Affiliations:** 1 State Key Laboratory of Pharmaceutical Biotechnology and Nanjing Drum Tower Hospital, School of Life Sciences, Chemistry and Biomedicine Innovation Center, Nanjing University, 163 Xianlin Avenue, Nanjing 210023, Jiangsu, China.; 2 Institute for TCM-X, MOE Key Laboratory of Bioinformatics/Bioinformatics Division, BNRIST, Department of Automation, Tsinghua University, Beijing 100084, China.; 3 School of Pharmaceutical Science and Yunnan Key Laboratory of Pharmacology for Natural Products, Kunming Medical University, Kunming 650500, China.

## Abstract

Emerging evidence has linked high dietary fructose intake with adverse health outcomes, yet its role in colitis-associated colorectal cancer (CAC) remains underexplored. Here, we demonstrate that high fructose consumption aggravates intestinal inflammation and significantly promotes tumorigenesis in an AOM/DSS-induced CAC model via activation of the EREG-EGFR signaling axis. We first developed the Food∩TCM database, which encompasses 112 medicine and food homologous TCM, along with their chemical compositions. Using the network-based pharmacological intelligence platform, we identified *Laminaria japonica* (kelp) and its bioactive components—fucoidan and dieckol—as promising dietary interventions. Dieckol was found to target the RNA/DNA-binding protein CNBP, which has traditionally been regarded as a positive regulator of pro-inflammatory gene expression, but here was shown to enhance its nuclear localization under inflammatory conditions and thereby repress *Ereg* transcription. Notably, fructose promoted O-GlcNAcylation-mediated stabilization and secretion of EREG in inflammatory fibroblasts, contributing to tumor progression. Together, these findings reveal the tumor-promoting effects of dietary fructose in CAC and highlight CNBP as a key regulator restraining *Ereg* expression. Our findings underscore the significance of integrating food-based strategies into chronic disease prevention, and support the development of *Laminaria japonica*-derived compounds as safe, long-term dietary agents for managing colitis-associated colorectal cancer.

## Introduction

Nutritional intake has a significant impact on overall health, and dietary interventions are commonly employed in the treatment of some diseases 1. An increasing body of evidence suggests that the intake of specific amino acids and carbohydrates can influence the progression and treatment outcomes of cancer, particularly colorectal cancer 2,3. Colorectal cancer (CRC) is one of the most common malignancies, clinically categorized into colitis-associated colorectal cancer (CAC) and sporadic colorectal cancer (SCRC) 4. The incidence of colorectal cancer varies significantly, with differences of 6-8 times across various countries and regions, reflecting socio-economic development 5. Genetic and environmental risk factors play crucial roles in the pathogenesis of colorectal cancer. Factors such as dietary patterns, obesity, and lifestyle contribute to an increased risk of colorectal cancer 6.

In contrast to sporadic colorectal cancer, colitis-associated colorectal cancer primarily occurs in patients with inflammatory bowel disease (IBDs), where prolonged exposure to chronic inflammation makes the disease course more susceptible to dietary influences and other factors 7. Fructose is a natural monosaccharide found in foods such as fruits and honey, widely consumed due to its high sweetness, low glycemic index, and favorable flavor profile. However, recent scientific research has revealed that a high-fructose diet may be a significant factor in the rising incidence of diseases such as diabetes, inflammatory bowel disease, non-alcoholic fatty liver disease, cardiovascular diseases, and colorectal cancer 8,9. Consequently, the impact of fructose on various diseases, particularly the progression of chronic diseases, requires further investigation. The connection between fructose and gut diseases, particularly colitis-associated colorectal cancer, remains unclear.

Medicine-food homologous substances refer to natural substances that possess both medicinal and nutritional properties, coexisting harmoniously within a single entity 10. Due to their unique biological activities and relatively low toxicity, they exhibit significant research value and economic potential 11,12. For instance, honey exhibits antioxidant, anti-inflammatory, and anticholinesterase activity and alleviates multiple neurodegenerative diseases^13^,^14^. Goji berries, renowned for their antioxidant properties, enhance immunity and maintain homeostasis 15. In recent years, modern science has increasingly recognized the validity of medicine-food homologous. Research has begun to unravel the bioactive compounds in these foods, providing empirical support for their health benefits. This alignment with global health trends, such as the rise of preventative medicine and natural therapies 16. Thus, medicine-food homologous bridge the gap between nutrition and pharmacology, offering a holistic approach to health that is both accessible and effective. It encourages a lifestyle where everyday meals are imbued with healing potential. However, overcoming the challenges related to research, regulation, and public education is crucial to fully harnessing its potential in contemporary healthcare systems 17. Herein, we demonstrate the promoting effect of high-fructose diet on colitis-associated colorectal cancer, revealing the potential adverse impacts of dietary patterns on physiological systems. Concurrently, we established a database named Food ∩ TCM which integrated the basic information as well as chemical profiles of 112 known medicine-food homologues and identified kelp (*Laminaria japonica*) as a candidate intervention for colitis-associated colorectal carcinogenesis through a network-based genome-wide drug-target prediction algorithm 17.

Furthermore, we elucidated the critical role of Dieckol—a chemical compound from kelp—in suppressing EREG transcription and secretion via targeting CNBP. Cellular nucleic acid-binding protein (CNBP), also called zinc-finger protein 9 (ZNF9), this DNA- and RNA-binding protein, with broad sequence specificity, is associated with a variety of cellular functions, including transcription and translation 18. Traditionally, CNBP has been characterized as a positive regulator of pro-inflammatory cytokines such as IL-6 and IL-12, thereby contributing to immune regulation 19, 20. Nevertheless, whether CNBP exerts functions within parenchymal cells in response to microenvironmental inflammatory cues, particularly in maintaining tissue homeostasis, has remained largely unexplored. Here, we found that upon stimulation of fibroblasts with the inflammatory cytokine TNF, CNBP translocated into the nucleus and controlled the transcriptional expression of EREG, thereby preventing malignant tissue over-proliferation. Our results propose an innovative framework or target for investigating the bidirectional health impacts of dietary choices, offering novel prevention and therapeutic strategies for colorectal cancer management.

## Methods

### High-fructose exacerbated DSS-induced colitis model and colitis-associated cancer (CAC) model

Eight-week-old male C57BL/6 mice were obtained from GemPharmatech Co. Ltd. and housed under controlled conditions (21±3°C, 12-hour light/dark cycle). All experiments were approved by an Institutional Animal Care and Use Committee (IACUC) at the Model Animal Research Center of Nanjing University (IACUC2506005). For the high-fructose exacerbated colitis model, thirty 8-week-old male C57BL/6 mice were randomly divided into three groups. The Fructose+DSS group received drinking water supplemented with 30% fructose, which was refreshed every 48 hours for 4 consecutive weeks. The remaining groups consumed standard drinking water. At week 5, 2.5% DSS was administered in the drinking water of both DSS and Fructose+DSS groups, with the latter maintaining 30% fructose supplementation. On day 6 post-DSS administration, the DSS solution was replaced with either standard water or 30% fructose water. Body weight, fecal consistency, and anal hygiene status were monitored throughout the experiment. All mice were euthanized on day 8 post-DSS initiation following a 12-hour fasting period.

For the CAC model, the mice received a single intraperitoneal injection of 7.5 mg/kg AOM solution on day 0. The AOM/DSS+Fructose group was provided with 30% fructose-supplemented drinking water continuously throughout the entire modeling period. On day 5, both AOM/DSS and AOM/DSS+Fructose groups were administered 2.5% DSS in drinking water for 5 consecutive days. This was followed by a 14-day recovery period starting, during which mice received either standard water or 30% fructose water. The DSS challenge-recovery cycle was repeated twice. Body weight, fecal consistency, and anal health were monitored throughout the experiment. Tissues were harvested for analysis at the inflammatory phase (tumor initiation, day 30) and adenoma phase (day 75). At the experimental endpoint, colons were opened longitudinally and visible tumors (≥1 mm in diameter) were counted. Tumor load was determined by summing the calculated areas of all identified tumors for each mouse. Tumor length and width were measured using an electronic caliper, and tumor area was calculated accordingly. All measurements were performed in a blinded manner.

To evaluate the ameliorative effects of the selected medicinal-food homologous compounds on high-fructose diet-aggravated colitis-associated colorectal cancer, mice were administered daily via intragastric gavage with kelp extract (200 mg/kg), dieckol (100 mg/kg), and fucoidan (100 mg/kg). According to previous reports, the disease activity index (DAI) was determined based on three parameters: weight loss, stool consistency, and the severity of intestinal bleeding.

### Kelp extract

First, thoroughly wash dried kelp (*Laminaria japonica*) with abundant distilled water to remove surface dust, salts, and soluble impurities. Kelp fragments were mixed with distilled water, followed by the addition of enzymatic agents (pectinase and cellulase). The suspension was stirred at 50°C for 24 hours, subsequently centrifuged at 3000 g for 20 minutes at 4°C, and vacuum-filtered. Three volumes of 60% ethanol were then added for precipitation. After 18 hours of extraction, the solution was filtered, concentrated using a rotary evaporator, and finally spray-dried to obtain the powdered extract 21.

### Cell culture

L929 cells were cultured in DMEM (11965092, Gibco) supplemented with 10% fetal bovine serum (10100147, Gibco) and 1% penicillin-streptomycin (C0222, Beyotime); NIH-3T3 cells were maintained in DMEM containing 10% newborn calf serum (26010074, Gibco). Both cell lines were incubated at 37°C with 5% CO2.

### Single-cell RNA sequencing

Murine colon tissues were collected and generate single-cell gel bead in-emulsions (GEMs) using the 10× Chromium platform according to the manufacturer's protocols. Subsequent library construction and sequencing were performed. All libraries underwent paired-end sequencing with dual indexing on the NextSeq 500 platform (Illumina).

### Data preprocessing

Raw scRNA-seq data were processed by Cell Ranger (v.3.1.0) software (10× Genomics) for read alignment, barcode assignment and unique molecular identifier (UMI) counting, based on genomes (mm10-3.0.0). Cells meeting criteria of UMI >200 and mitochondrial RNA <10% were retained. Subsequent analyses were performed using the Seurat package in R. Quality-controlled single-cell data were first integrated and subjected to principal component analysis (PCA) for linear dimensionality reduction. A K-nearest neighbor (KNN) graph was then constructed to refine cellular neighborhood relationships based on edge weights. Proximity distances were calculated using FindNeighbors, followed by iterative clustering with FindClusters. Finally, the dataset was visualized through Uniform Manifold Approximation and Projection (UMAP) for non-linear dimensionality reduction. Cell clustering was conducted via FindClusters function using the top 30 principal components (PCs) to resolve cell identities. Cluster-specific marker genes were identified using the Wilcoxon rank-sum test (FindAllMarkers, default parameters) and annotated based on known markers and top-ranked genes.

### Pseudotime analysis

Monocle2 was applied to illustrate the developmental epithelial cells trajectories using the UMI count matrix. Cluster-specific variable genes were employed to order cells along the pseudotemporal axis. The epithelial cells cluster 3 and 8 (E03 and E08) was defined as the root state.

### CellPhoneDB analysis

The analysis starts with input preparation, a single-cell expression matrix with cell-type annotations and gene symbols. Next, mean expression levels of ligands and receptors are calculated for each cell type. Finally, interaction scores are computed by multiplying the average ligand expression in a source cell type with the average receptor expression in a target cell type, yielding a quantitative measure of potential communication strength between cell populations.

### Medicine and food homologous TCM screening for high-fructose exacerbated CAC

Food∩TCM is the first and most comprehensive database in China that integrates fundamental information on the medicinal sources and uses of medicine-food homologous substances with their chemical and nutritional compositions (http://101.200.131.49:8000/). Users can browse or search the database based on personal preferences, either by the name of the medicinal substance (hereafter referred to as “herb”) or by the compound name. Future versions will support additional search options, including the source of the herb as well as its descriptions and functional annotations.

The current version of Food∩TCM (v1.0) integrates data from multiple authoritative sources, including FooDB, FooDisNET, TASLY-TCM, PubChem, PubMed, the Chinese Pharmacopoeia (2020 edition), and the Chinese Food Composition Table (Standard Edition, 6th Edition). This database serves as a valuable reference and guiding resource for researchers studying medicine-food homologous substances in Traditional Chinese Medicine and for the development of related health products.

To identify targeted food with potential therapeutic effects against the key mechanisms of high-fructose exacerbated CAC, we systematically compiled data on medicine-food homologous TCM and their compound compositions from the literature and various databases. Additionally, we established an online database, Food∩TCM, to facilitate further exploration and application of these findings. In total, 112 herbs and their related compound were documented. Before screening, the data was further processed by being annotated to PubChem database and TCM with at least one valid compound in PubChem were kept for further analysis. The compounds in these homologous were then transferred into chemical similarities between reference compounds in a network-based whole-genome drug-target prediction algorithm 17. And the biological effect profile of every compound in each medicine-food homologous TCM was calculated by this algorithm. Further, according to our previous statistical strategy 22, the holistic targets of each medicine-food homologous TCM were obtained (adjust P value < 0.05, Benjamini-Hochberg procedure).

With the four gene sets obtained from scRNA-seq of high-fructose exacerbated CAC and predicted holistic targets of 101 medicine and food homologous, the closeness between each gene set and each medicine-food homologous TCM was estimated on the hypergeometric distribution. The candidate medicine and food homologous for high-fructose-induced CAC was screened based on the significance and mapping count. The expression pattern of kelp in scRNA-seq was measured by the mean expression of the holistic targets of kelp in different cell types and visualized by VlnPlot function from Seurat package in R.

### Key compound identification from kelp

The biological effect profiles of compounds in kelp were kept for further analysis. In total, 50 compounds were identified in kelp according to our collection. And the druglikeness of these compounds were calculated by the implementation based on Python rdkit package 23. 27 compounds with quantitative estimate of drug-likeness (QED) score no less than 0.3 were kept for further analysis. Additionally, the same as screening TCM for high-fructose exacerbated CAC, the closeness between each gene set and every compound in kelp was also estimated on the hypergeometric distribution. The expression pattern correlations of these compounds and kelp were estimated on the Spearman correlation based on the corresponding expression on each cell belonging to stromal cells or epithelial cells, visualized by ggplot2 package in R. The network similarity of two gene sets was implemented by GOSemsim package in R, with methods set as “Wang”.

### Network target of kelp in the treatment of high-fructose exacerbated CAC

In order to find the key mechanism of kelp in treating high-fructose-induced CAC, network target analysis was performed based on the holistic targets of kelp and the four gene sets. The biological network composed of certain molecules in gene set A, B, C, D, as well as the holistic targets of kelp and CRC-related biomolecules were constructed based on protein-protein interaction data, and were then visualized by Cytoscape. KEGG enrichment was conducted to find the significantly enriched pathways in either kelp or the four gene sets, implemented by clusterProfiler package in R. Pathways significantly enriched by the holistic targets of kelp were then categorized into 4 modules, including Metabolism, Environmental Information Processing, Cellular Processes and Organismal Systems. On the other hand, 10 pathways were found to be significantly enriched by the molecules in all four gene sets, and 9 of them were potentially targeted by kelp intervention (adjust P value < 0.05, Benjamini-Hochberg procedure). The pathways were visualized using the R package ggplot2, and the -log10 of the enrichment significance (P value) was standardized for each gene set to observe more obvious differences.

The correlations of the expression of EGFR and EREG between kelp, Fuc and dieckol were estimated on the Spearman correlation on each cell belonging to the whole detected cells, stromal cells or epithelial cells, respectively. And the expression levels of EREG and EGFR in scRNA-seq were measured and visualized by VlnPlot function from Seurat package in R, in different cell types.

### H&E staining

Colon tissues were fixed in 4% paraformaldehyde for 24 hours, followed by paraffin embedding and sectioning. Paraffin sections were deparaffinized in xylene after 90-minute incubation at 65°C, then rehydrated through descending concentrations of ethanol. Sections were rinsed in distilled water before staining with hematoxylin for 1 minute. Differentiation was performed using 1% hydrochloric acid-ethanol, followed by bluing in 1% ammonia water. After rinsing, sections were counterstained with 1% eosin and mounted with neutral balsam for microscopic examination.

### Immunohistochemistry

Following deparaffinization and antigen retrieval, sections were permeabilized with 0.5% Triton X-100. Endogenous peroxidase activity was blocked, and non-specific binding was prevented using 3% goat serum. Primary antibodies were incubated at 4°C overnight, followed by species-specific secondary antibodies at room temperature for 2 hours. After PBS washes, DAB was applied, and the reaction was stopped with double-distilled water. Hematoxylin counterstaining was performed prior to mounting. All steps were optimized to ensure specificity and reproducibility.

### Immunofluorescence staining

Following antigen retrieval, tissue sections or cells were permeabilized with 0.5% Triton X-100 for 1 minute, then washed with PBS on a rocking platform. After blocking non-specific binding sites, species-specific primary antibodies were applied and incubated overnight at 4°C. On the following day, sections were allowed to equilibrate to room temperature for 30 minutes. Corresponding fluorescent secondary antibodies (selected based on host species of primary antibodies) were subsequently incubated in the dark for 2 h, followed by three PBS washes. DAPI working solution (1:5000 dilution) was applied for nuclear counterstaining at room temperature for 30 minutes, then removed by washing. Finally, sections were mounted with antifade mounting medium to preserve fluorescence signals.

### Microscale thermophoresis (MST)

NIH-3T3 cells were transfected with GFP-CNBP plasmids or mutant plasmids and lysed 48 hours post-transfection. Dieckol, utilized as a ligand, was diluted twofold and combined with an equal volume of cell lysate to yield a final fluorescence intensity of 400. Binding assays were conducted using the Monolith NT.115 instrument, followed by quantitative analysis of protein-ligand interactions via MO.Affinity Analysis software.

### ChIP assay

NIH-3T3 cells were transfected with GFP-CNBP plasmids and, 48 hours post-transfection, were subjected to appropriate stimulatory conditions. Following transfection, chromatin immunoprecipitation was performed. Briefly, target proteins and DNA were crosslinked using 1% formaldehyde. Cells were collected, lysed, and genomic DNA was fragmented via sonication. Reverse crosslinking was then conducted to assess the size distribution of sheared DNA fragments. After optimizing sonication conditions, chromatin immunoprecipitation was performed using Protein A+G Agarose/Salmon Sperm DNA beads. Enriched DNA fragments were subsequently quantified by qPCR. Primers were designed based on the promoter sequence of the *Ereg* gene and chromatin accessible regions identified using the Cistrome Data Browser, ensuring targeted amplification of regulatory elements.

### Cellular thermal shift assay

L929 cells were equally divided into two 10 cm dishes and cultured overnight. One dish was treated with dieckol (10 mM), while the other received 0.1% DMSO as a control. After 2 hours, cells were harvested, aliquotted into ten 200 μL PCR tubes, and subjected to heat treatment in a thermocycler. Samples were centrifuged at 13,000 × g for 20 minutes, and analyzed by Western blotting to detect target proteins.

### Plasmid DNA transfection

NIH-3T3 cells were seeded into culture plates and transfected with GFP-CNBP, EREG plasmids, or corresponding vectors. All plasmids were sourced from Corues Biotechnology. Lipofectamine™ 3000 were purchased from Thermo Fisher Scientific.

### ELISA assay

The protein level in mice serum was detected by an ELISA kit (ELK Biotechnology). Following initial incubation for 1.5 hours, the samples were washed, and the supernatant was discarded. The detection antibody was then added, followed by a secondary 1.5-hour incubation. After incubation with TMB substrate and stop solution, absorbance at 450 nm was measured to quantify EREG protein levels.

### Cell counting kit-8 assay

Cell viability was assessed using the Cell Counting Kit-8 (CCK-8, MCE) following the manufacturer's protocol. NIH-3T3 cells were seeded in culture plates for 12 hours to transfection with the indicated EREG plasmid. After 48 hours, varying concentrations of fructose or OSMI-1 were added to the culture medium and incubated for an additional 24 hours. The supernatant was collected, centrifuged at 1000 × g to remove cellular debris, and then incubated with MC38 cells for 24 hours. CCK-8 solution was added to the reaction mixture, and the absorbance was measured at 450 nm using a microplate reader.

### Dual luciferase reporter gene assay

NIH-3T3 cells were seeded in a 96-well plate and transfected with the indicated plasmids or vectors. After 48 hours, 100 μL of cell lysis buffer was added to each well. Following supernatant collection, 100 μL of firefly luciferase substrate was added to measure relative light units (RLU). Subsequently, 100 μL of Renilla luciferase detection reagent was added to determine RLU. Using Renilla luciferase activity as control, the RLU value from firefly luciferase was normalized to that of Renilla luciferase. The resulting ratio was used to compare the activation levels of the reporter gene across different samples.

### TRAP analysis

To identify dieckol-binding proteins in L929 cells, we implemented the Target-Responsive Accessibility Profiling (TRAP) approach. Two independent cultures of L929 cells were treated with either 50 nM TNFα alone or 10 μM dieckol combined with 50 nM TNFα. Following a 2-hour incubation period, cellular membranes were permeabilized using M-PER buffer. The resulting lysates were subjected to covalent labeling via the addition of formaldehyde and borane pyridine complex, which specifically target lysine residues in proteins under ambient conditions for accessibility profiling. Detailed methodological protocols are provided in previously published literature.

### EREG degradation analysis

L929 cells were cultured in serum-free medium supplemented with 50 μg/mL cycloheximide for 1 hour. Subsequently, cells were treated with 10 mM fructose and incubated for 3, 6, or 9 hours. Following incubation, cells were lysed, and total protein extracts were subjected to Western blotting analysis to assess target protein expression levels.

### Statistical analysis

The data represent the mean ± SEM, Unpaired two-tailed Student's t tests and One-way analysis of variance (ANOVA) with Tukey multiple comparison tests were used to assess statistical significance. Statistical significance was set at *P<0.05, **P<0.01, ns, not significant.

## Results

### A high-fructose diet exacerbates colitis-associated colorectal cancer in mice

**Figure 1:**
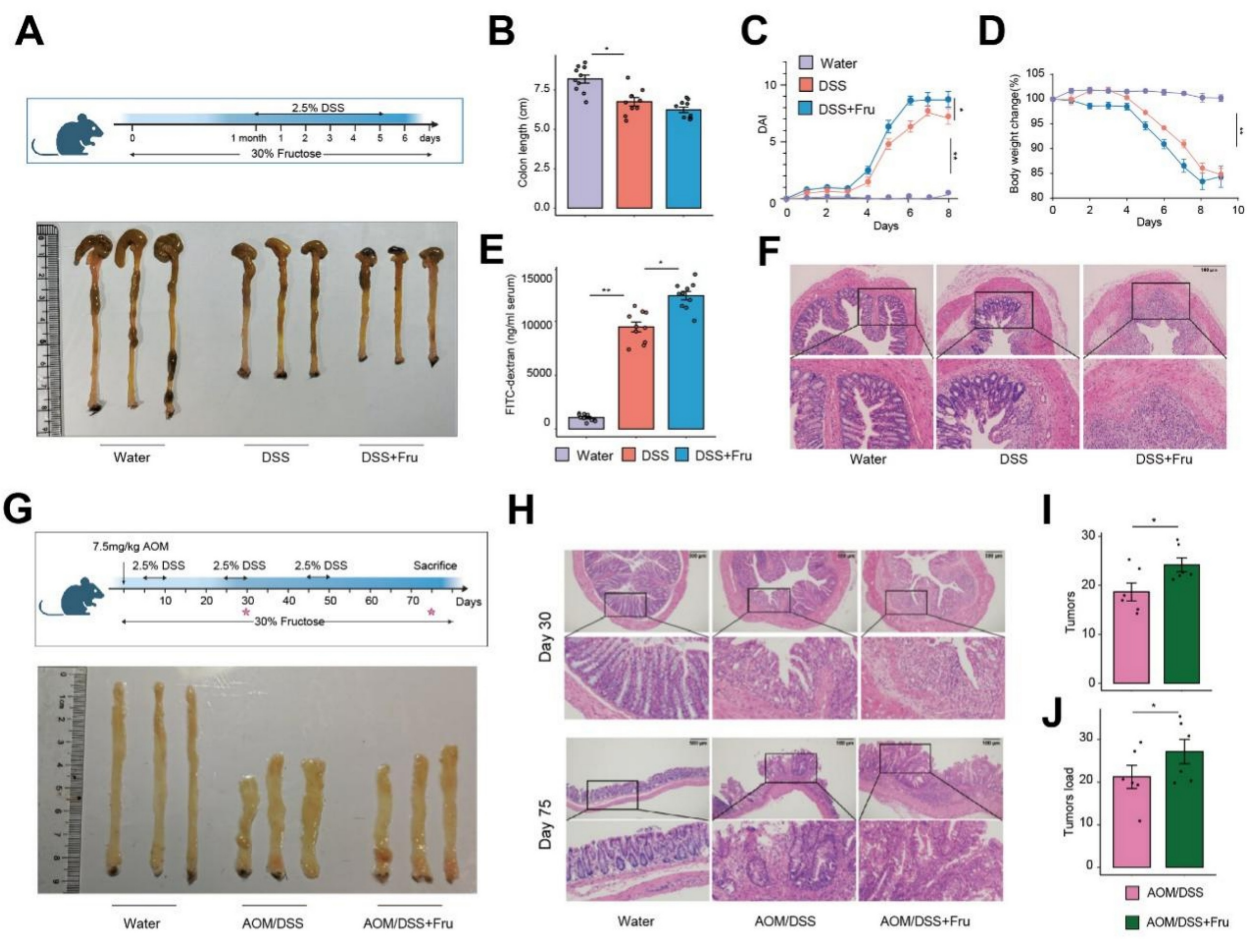
** Fructose-rich nutritional intervention promotes neoplastic progression in colitis-associated colorectal cancer. A.** Schematic overview of experimental design and appearance of colon. **B-D.** Intestinal length (B), DAI evaluation (C) and weight loss (D) in the water group, DSS group and DSS + Fru treatment group (n = 10). **E.** Serum FITC-dextran concentrations were analyzed (n = 10). **F.** The H&E staining of colon tissues. **G.** Experimental flow chart and appearance of colon. **H.** Representative H&E staining of colon tissues at 30 and 75 days after the beginning of DSS challenged. **I-J.** Tumor numbers (I) and tumor load (J) of colons from mice treated with AOM/DSS or AOM/DSS + Fru.

Accumulating evidence links fructose consumption to systemic inflammation 9. To elucidate the effects of a high-fructose diet on the progression of colitis, we utilized a dextran sulfate sodium (DSS)-induced mouse model, providing drinking water containing either 30% fructose or drinking water for 7 days (**Figure 1A**). We observed that the DSS + Fru mice exhibited significant aggravation in shortening of rectum length, disease activity index (DAI) and body weight loss compared with mice treated with DSS alone (**Figure 1B-D**). Furthermore, the levels of intestinal permeability, histological damage, inflammatory factors and abdominal reactive oxygen species were all elevated in the DSS + Fru mice compared to the DSS group (**Figure 1E-F and S1A-B**). To further explore the involvement of fructose in tumorigenesis, we treated mice with azoxymethane (AOM), a chemical carcinogen, followed by multiple cycles of DSS (**Figure 1G**). On day 30 of the experimental model, corresponding to the inflammatory phase of intestinal pathogenesis before the development of mature adenomas, our findings revealed that a high-fructose dietary regimen significantly intensified intestinal inflammatory responses (**Figure 1G-H and S1C-E**). Substantially, more raised macroscopic polyps and precancerous lesions and markedly increased tumor load in colons of AOM/DSS + Fru mice were observed (**Figure 1G-J**). These data suggest that high fructose intake exacerbated the progression of colitis-associated adenomas in mice.

### Single-cell RNA sequencing identifies multiple cell types in high fructose-accelerated CAC tumorigenesis

**Figure 2:**
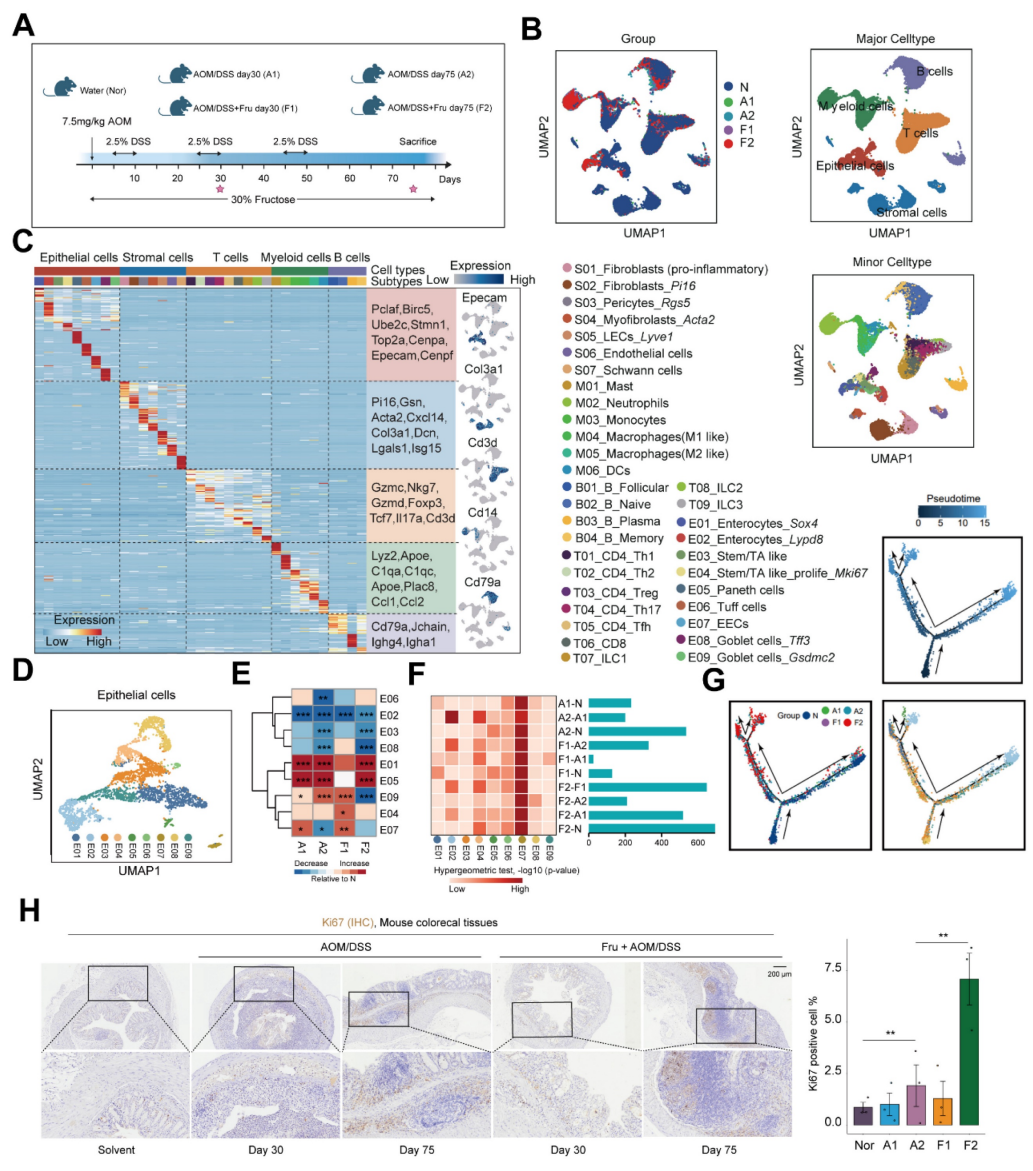
** Single-cell transcriptomic map of high fructose-accelerated CAC mice. A.** Schematic of mice colon tissue collection. **B.** UMAP plots showing the distribution of cell types by groups. **C.** Heatmap showing representative marker genes across cell types. **D.** UMAP plot showing the subtypes of epithelial cells. **E.** Heatmap showing proportion of indicated subtypes compared to Nor. Chi-square test was employed to assess statistical significance. **F.** Comparison of group-specific DEG and cell subtype-specific marker genes. Heatmap (left) shows the significance of overlap between group-specific DEG (rows) and cell subtype-specific marker genes (columns), bar plot (right) displays the number of DEG per comparison.** G.** Pseudotime trajectory of epithelial cells subtypes by Monocle2. Trajectory is colored by pseudotime, group, and cell subtypes. **H.** Ki67 staining of colon tissues obtained from the indicated mice. Ki67^+^ cell were quantified using ImageJ.

To further elucidate the mechanisms by which high-fructose intervention promotes colorectal cancer, we conducted single-cell RNA sequencing on colon tissue collected from mice subjected to various treatments. Specifically, we obtained colon tissues from mice challenged with water (Nor), AOM/DSS at 30 and 75 days (A1, A2) and AOM/DSS combined with fructose supplementation at 30 and 75 days (F1, F2) (**Figure 2A and 2B**). Colorectal cancer, which originates from polyps formed by epithelial lesions, involves a complex mechanism wherein the specific role of epithelial cells in CAC progression remains poorly understood. Therefore, we divided the epithelial cells into eight subsets (E01-E09) (**Figure 2C and 2D**). Compared to the Nor, the proportions of the subsets E01, E04, and E05 increased in the A1, A2, F1, and F2 (**Figure 2E and S2A-B**). Notably, we found that the Differentially Expressed Genes (DEG) of E04 and E07 had a high overlap with the DEG involved in CAC progression, suggesting that the E04 and E07 program plays an important role in high fructose remodeled epithelial cell niche (**Figure 2F**). To further elucidate the developmental and functional dynamics of epithelial cells subtypes, we conducted a pseudotime trajectory analysis. The results showed that E01, E02, E04 and E05 were localized at different ends of the trajectory (**Figure 2G**). These data highlight the crucial role of E04_ Stem/TA like -*Mki67* in high-fructose-promoted CAC progression. To validate the predicted function of E04, we performed staining for Ki67, and the results showed that Ki67-positive cells increased in other mice compared to the Nor group, particularly in the F2 mice (**Figure 2H**).

### Dynamic crosstalk between epithelial and stromal cells orchestrates the progression of CAC

**Figure 3:**
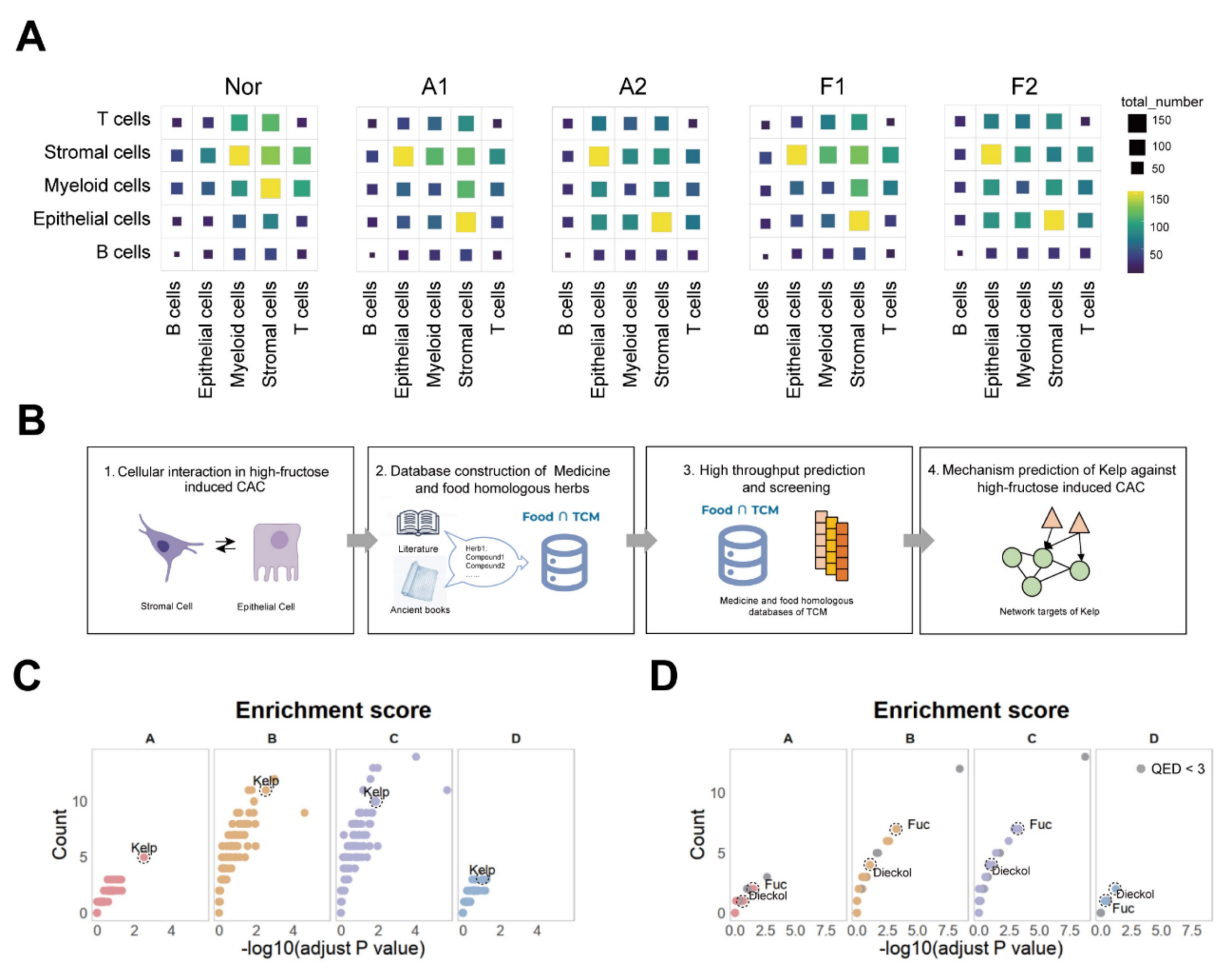
** Screening identified the kelp as capable of improving the progression of CAC. A.** Cell-cell interactions among cell types in indicated groups. **B.** Schematic overview of screening design.** C.** Dot plot visualization of medicinal-food homologous screened by the network-based whole-genome drug-target prediction algorithm. **D.** Dot plot visualization of kelp ingredients screened by the network-based whole-genome drug-target prediction algorithm.

During colorectal cancer progression, tumor microenvironment interactions contribute to tumor evolution and expansion. To delve deeper into the intercellular communication in CAC, we analyzed cell-cell interactions in different groups. During this process, the epithelial-stromal interaction was notably increased (**Figure 3A**). Similarly, we categorized the stromal cells into seven subtypes to map out the detailed cell communication networks between stromal cells and the epithelial cells (**Figure S3A-C**). We found that the cell communication produced by the E04 program significantly increased with fructose intake and adenoma formation, particularly in relation to S01_Inflammatory fibroblasts (**Figure S4**). We next wanted to seek overexpressed ligand-receptor pair (s) in the interaction between epithelial cells and stromal cells that may drive the development of CAC (**Table S1**). These results suggest that epithelial-stromal interactions may play a pivotal role in high-fructose-driven CAC tumorigenesis.

### Screening for medicine-food homologous that can prevent CAC adenomas

We wanted to leverage these ligand-receptor pairs to develop strategies for preventing and treating CAC tumorigenesis. We aim to systematically analyze these ligand-receptor pairs based on specific patterns. By comparing the A2 stage with the A1 stage, the F1 stage with the A1 stage, the F2 group with the F1 group and F2 stage with the A2 stage we identified Gene Set A, which represents ligand-receptor pairs that change specifically during the transition from colitis to CAC under high-fructose conditions. Next, comparing the F1 with the N, and the F2 with the N, we derived Gene Set B, which reflects ligand-receptor pairs altered during the inflammation-to-tumor transition under fructose conditions. By comparing the A1 stage and A2 stage to the N group, we identified Gene Set C, representing ligand-receptor pairs involved in the colitis-to-cancer progression. Finally, by subtracting the ligand-receptor pairs in Gene Set C from those in Gene Set B, we derived Gene Set D, which highlights ligand-receptor pairs influenced by fructose during the colitis-to-cancer transition (**Figure S5**). Collectively, these four gene sets represent the various ways in which fructose impacts the progression of CAC.

We first developed the Food∩TCM database, which encompasses 112 medicine and food homologous TCM, along with their chemical compositions, to identify potential candidates medicine-food homologous for preventing CAC tumorigenesis (**Figure 3B**). After filtering, 101 medicine-food homologous were selected, and 4,197 unique compounds were identified and annotated in the PubChem database, which were retained for further analysis (**Figure S6A and S6B**). To identify potential medicine-food homologous and key compounds for mitigating fructose-induced CAC, a series of network target-based computational predictions were employed to predict the holistic targets of each medicine-food homologous 17,23. Based on the enrichment result between these holistic targets and the four gene sets, the screening results revealed that kelp (*Laminaria japonica*) showed significant enrichment across all four effect gene sets, suggesting its potential in improving CAC tumorigenesis (**Figure 3C**). kelp, a ubiquitous marine macroalgae, is highly valued as a functional food source due to its rich profile of polysaccharides, vitamins, and mineral nutrients 24. These multifaceted health-promoting attributes position kelp as a promising candidate for both preventive healthcare and therapeutic interventions. At the cellular level, the holistic targets of kelp exhibited higher expression in stromal and epithelial cells (**Figure S6C**).

Natural substances have historically posed challenges in pharmacological mechanism research due to their complex compositions. To better elucidate the mechanism by which kelp mitigates CAC tumorigenesis, we utilized the UNIQ system to predict the monomeric components of kelp 25, 26. Our analysis identified 27 compounds with high drug-likeness out of the 50 compounds recorded in kelp (**Figure S7A**) 25, 27. Among these, Dieckol, a compound approved for marketing in the European Union, was significantly enriched in effect gene sets A, B, C, and D (**Figure 3D**). Additionally, another key compound in kelp, Fucoidan, also showed strong enrichment in effect gene sets A, B, and C, although it ranked lower in set D, likely due to the smaller number of genes in this set (**Figure 3D**). Beyond statistical-based screening, a network-based similarity evaluation revealed that the predicted targets of both dieckol and fucoidan demonstrated high network-based similarity across the four effect gene sets, as well as with the holistic targets of kelp, highlighting their pivotal roles in the overall therapeutic effects of kelp (**Figure S7B**) 22,28. At the cellular level, the holistic targets of Dieckol demonstrated significantly elevated expression in stromal cells, epithelial cells, and myeloid cells. In contrast, the targets of Fuc were more concentrated in stromal cells and myeloid cells (**Figure S7C**). Additionally, the expression profiles of kelp exhibited robust correlations with the predicted targets of both Dieckol and Fuc specifically within stromal and epithelial cells (**Figure S7D**). These results suggest that dieckol and fucoidan may play critical roles in amplifying the effects of kelp on CAC tumorigenesis.

### Kelp supplementation attenuates colitis-associated colorectal cancer progression

**Figure 4:**
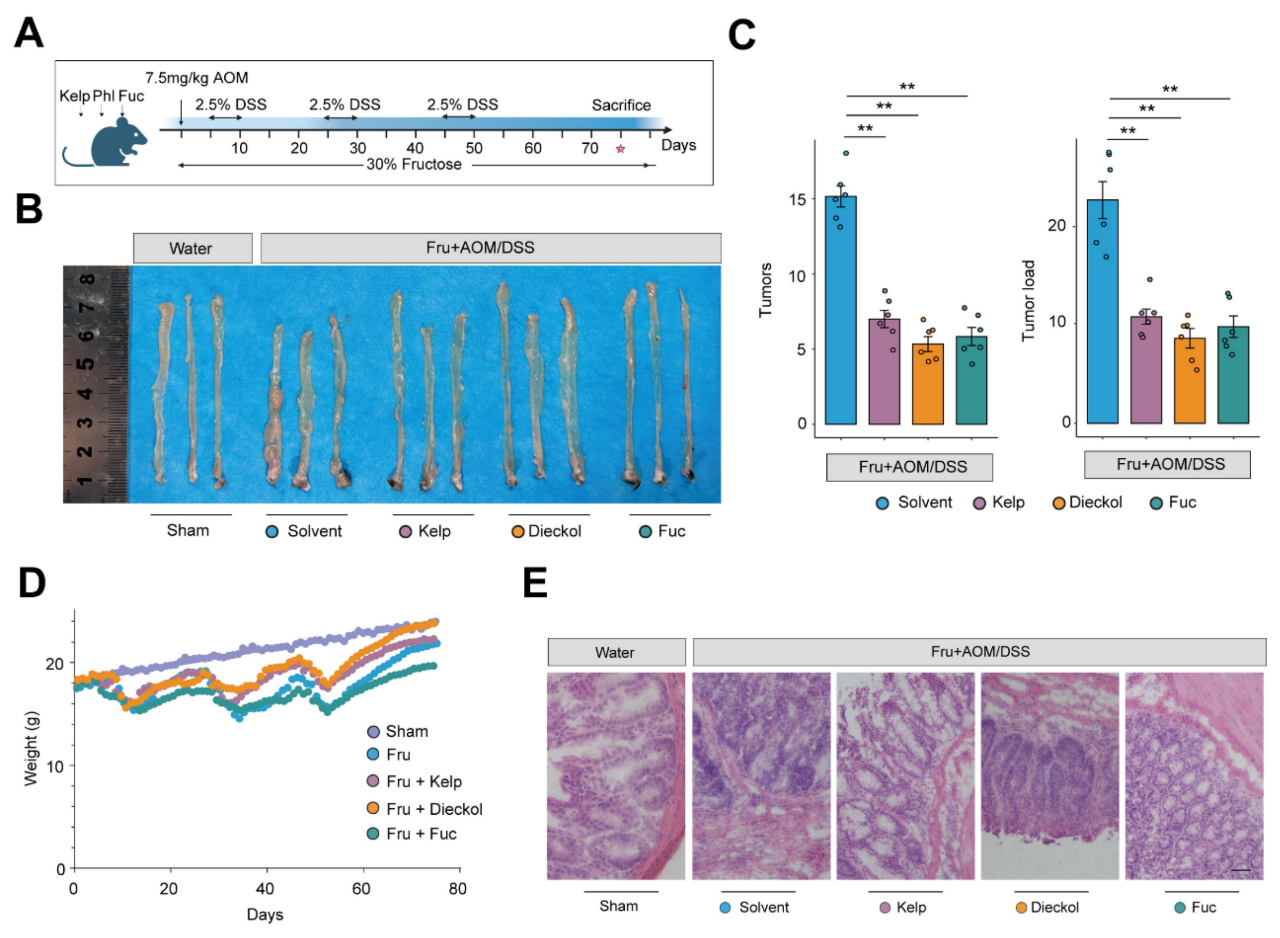
** Kelp, dieckol and fucoidan attenuates colorectal tumorigenesis in AOM/DSS model. A.** Schematic overview of experimental design. **B.** Representative colon specimens showing tumor development among the groups. **C.** Tumor numbers and tumor load of colons from mice. **D.** weight loss in different groups. **E.** The H&E staining in colon tissues. Scale bar = 50 μm.

To validate our screening framework, we employ the mouse model of high fructose-promoted CAC tumorigenesis with kelp extract, fucoidan, and dieckol** (Figure 4A)**. By day 75, an examination of the colonic tissue revealed that mice in the Fru group developed numerous and larger adenomas, primarily concentrated near the anal region. Remarkably, treatment with kelp, dieckol and fucoidan significantly reduced tumor size and, to some extent, decreased the number of adenomas **(Figure 4B and 4C)**. H&E staining of the colon tissues also showed that extensive damage to the crypt structure, villi, and mucus layer in the intestines of the Fru group, along with tumor formation. Consistently, treatment with kelp, dieckol and fucoidan preserved the mucus layer and crypt structure to some extent, resulting in fewer adenoma formations **(Figure 4D-4E)**. In summary, these results indicate that kelp, dieckol and fucoidan can significantly ameliorate high fructose-promoted CAC tumorigenesis.

### Kelp impairs tumor-promoting cell-cell interactions via disruption of the EREG-EGFR signaling axis

**Figure 5:**
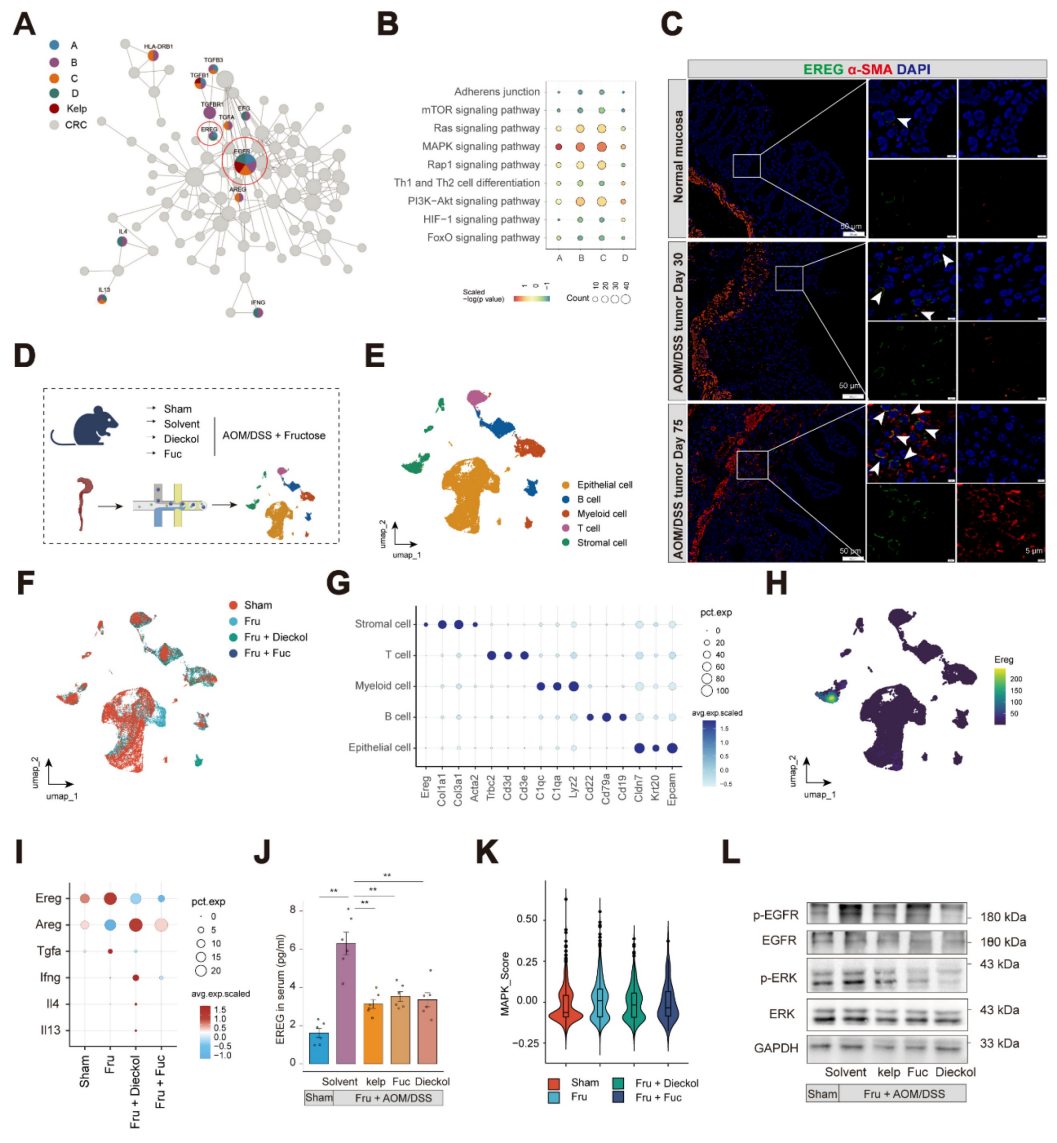
** Kelp impairs the EREG-EGFR signaling axis. A.** The network target analysis was performed based on the holistic targets of kelp and the four gene sets.** B.** KEGG enrichment was conducted to find the significantly enriched pathways in either the four gene sets or targeted by kelp. **C.** Immunofluorescence staining of colon with EREG (green), α-SMA (Red) and DAPI (blue). Scale bar: 50 μm or 5μm.** D.** Workflow of scRNA-seq analysis for colon tissue. **E-F.** UMAP plots showing the distribution of cell types **(E)** or groups **(F)**.** G.** Dotplot showing the expression of marker genes in cell types.** H.** Expression of *Ereg* in cell types. **I.** Expression of putative functional genes identified in Figure 5A across groups. **J.** EREG content in serum from indicated mice.** K.** MAPK pathway activity scores of MKI67⁺ epithelial cells in each group. **L.** Phosphorylation levels of EGFR and ERK expression of colon tissues from mice.

Utilizing the UNIQ network proximity analysis platform, we visualized the ligand-receptor interactions targeted by kelp and mapped the network proximity between drug targets and ligand-receptor pairs in a two-dimensional space. With colorectal cancer related targets as a reference, we aimed to identify the key targets of kelp. The analysis revealed that, in the context of colorectal cancer, EGFR ranked among the top targets and was present in all four previously identified effect gene sets, while TGFB1 appeared in the A, B and C sets. Additionally, the EGFR ligand EREG was significantly enriched **(Figure 5A)**. At the pathway level, the enrichment analysis results suggest that, the MAPK signaling pathway emerged as the most prominent across the four effect gene sets (**Figure 5B**). The epidermal growth factor receptor (EGFR) serves as a critical oncogenic driver in CRC, as its aberrant activation and subsequent downstream signaling cascades, such as MAPK, are fundamentally implicated in tumor development and progression^29^. Epiregulin (EREG), a ligand of EGFR, has been shown to facilitate CAC progression via activation of the ERK 30. Therefore, we hypothesize that kelp may promote tumor growth by modulating the MAPK pathway through the EREG-EGFR signaling axis.

Thus, we first investigated the distribution of EREG in colon tissue. At the single-cell level, *Ereg* are predominantly expressed in stromal cells and *Egfr* are widely expressed in stromal cells and epithelial cells (**Figure S8A-S8B**). At the level of stromal cell subsets, *Ereg* is expressed in subsets S01_ Fibroblasts(pro-inflammatory) and S04_ Myofibroblasts. Given that tumor-associated fibroblasts are the primary source of *Ereg*, we investigated the expression of EREG in αSMA^+^ cells located in the mucosal layer. We validated EREG localization to αSMA⁺ cells in the mucosal layer of the colon in both mouse models and clinical specimens, with its expression significantly increasing as the disease progresses (**Figure 5C and S8C-D**).

To further elucidate the mechanisms by which dieckol and fucoidan ameliorate colitis-associated adenoma progression, we performed single-cell RNA sequencing on colon tissues collected from mice before and after intervention (**Figure 5D-F**). The expression patterns of representative DEGs are illustrated in dot plots (**Figure 5G**). Similarly, *Ereg* was mainly expressed in stromal cells (**Figure 5H**). We further examined the pathways identified through the UNIQ network proximity analysis platform following dieckol and fucoidan intervention. Among these pathways, EREG showed the highest expression level, which was markedly reduced upon dieckol and fucoidan treatment (**Figure 5I**). Other ligands of the EGFR family were also examined, among which EREG exhibited the highest expression, and its level was reduced following dieckol and fucoidan intervention (**Figure S9A**). Notably, the treatment of kelp, dieckol, and fucoidan effectively inhibited EREG secretion in mouse serum (**Figure 5J**). Furthermore, circulating EREG concentrations showed a strong positive correlation with intestinal adenoma load (R = 0.76). Notably, both kelp supplementation and treatment with its bioactive compound resulted in a consistent downward shift in these correlated parameters, indicating a coordinated reduction in serum EREG levels and tumor burden (**Figure S9B**).

We next examined the colonic epithelial cells in mice and found that the MAPK pathway was activated in MKI67⁺ epithelial cells following AOM/DSS treatment, whereas this activation was suppressed in mice treated with dieckol and fucoidan (**Figure 5K and Figure S9C-E**). Consistently, our results showed that EGFR and ERK signaling were inhibited by the administration of kelp, dieckol, and fucoidan (**Figure 5L**). These findings indicate that kelp, dieckol, and fucoidan regulate EREG secretion by colonic αSMA^+^ fibroblasts, thereby potentially mediating their protective effects against fructose-promoted CAC tumorigenesis through inhibition of the EREG-EGFR-ERK signaling axis.

### O-GlcNAcylation induced by fructose facilitates stabilization of EREG

**Figure 6:**
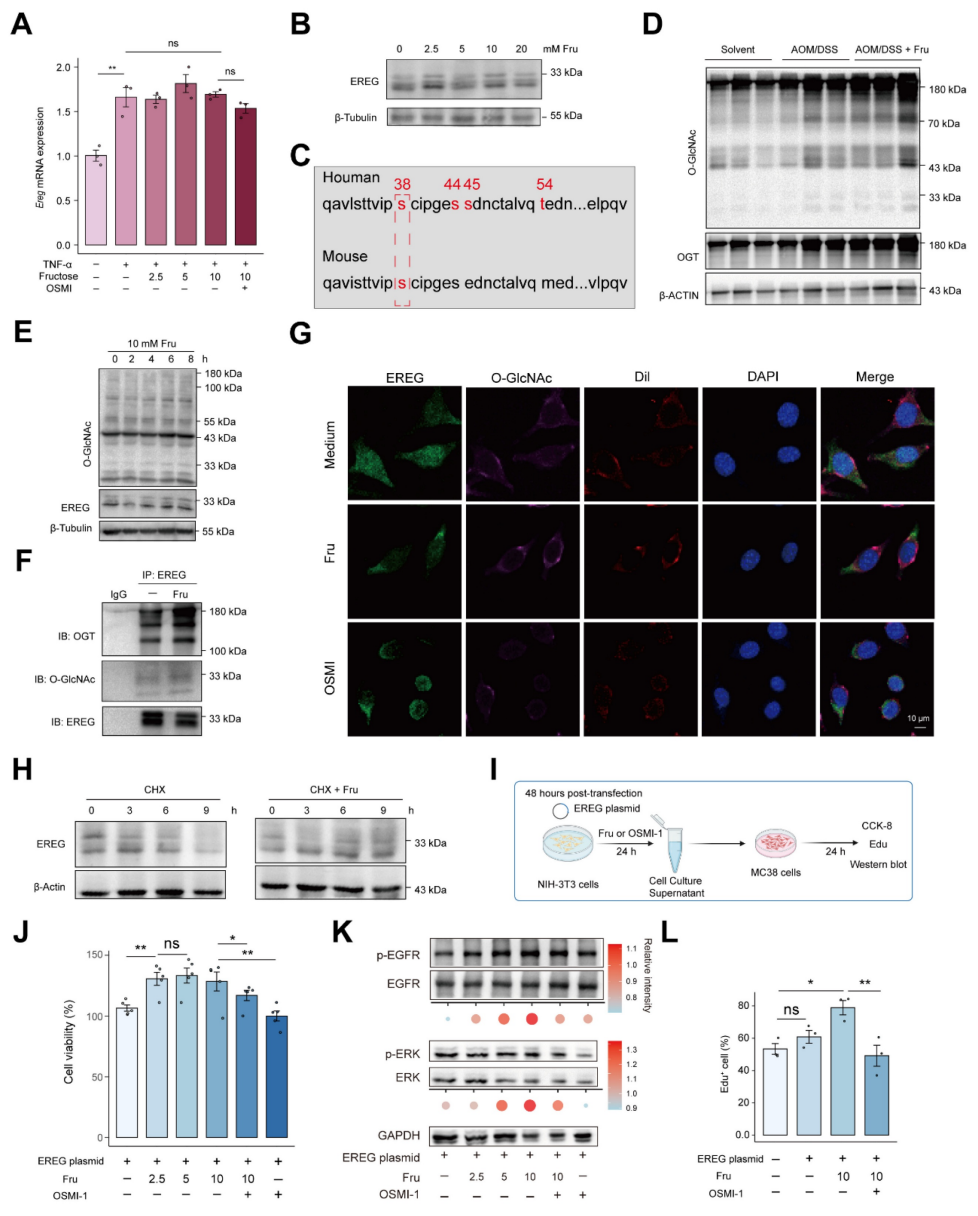
** Fructose enhances EREG stability via O-GlcNAcylation. A.** The expression of *Ereg* in response to TNF-α or different concentrations of fructose stimulation in L929 cells. **B.** The expression of EREG were analyzed by immunoblotting in L929 cells. **C.** Predicted O-GlcNAcylation amino acid residues of human and mouse EREG proteins by NetOGlyc4.0. **D.** The levels of O-GlcNAcylation and OGT were analyzed by immunoblotting in colon tissues from indicated mice.** E.** Western blot analysis of EREG and GlcNAcylation in L929 cells with fructose supplementation (10 mM) at the indicated times.** F**.L929 cells were treated with DMSO or fructose (10mM) for 24 h, and EREG was immunoprecipitated. EREG O-GlcNAcylation was analyzed by immunoblotting. **G.** Immunofluorescence analysis of colocalization between EREG, O-GlcNAcylation, and Dil membrane probe under indicated stimulation. Scale bar = 10 μm.** H.** L929 cells were treated with 10Mm fructose for 24 h followed by pulse-chase with 50 μg/mL cycloheximide. The expression of EREG were analyzed by immunoblotting at the indicated times.** I.** Experimental workflow for CCK-8, EdU, and immunoblotting **(J-K)**. OSMI-1 was applied at a concentration of 10 μM for 24 hours. **J.** MC38 cells viabilities were analyzed using CCK-8 assay after the L929 supernatant culture. **K.** Phosphorylation levels of EGFR and ERK of MC38 cells. Band intensities were quantified using ImageJ, and the resulting data were visualized and statistically analyzed using R. **L.** EdU were performed to analyze MC38 proliferation. EdU^+^ cells represent newly proliferating cells.

We next examined the mechanisms by which fructose accelerates CAC development. We supplemented L929 fibroblasts with fructose and examined the levels of EREG. Surprisingly, fructose supplementation did not alter the mRNA levels but increased the protein expression of EREG (**Figure 6A and 6B**). This suggests that post-translational modifications may be involved in the mechanism of fructose. As a fundamental monosaccharide, fructose can promote hepatocellular carcinoma progression by enhancing O-linked N-acetylglucosaminylation 31. O-linked N-acetylglucosaminylation (O-GlcNAcylation), a dynamic and reversible post-translational modification occurring on serine or threonine residues, is highly responsive to nutrient availability 32, 33. Thus, we explored whether the high-fructose diet promotes tumorigenesis in CAC through the O-GlcNAcylation of EREG.

Initially, we identified O-GlcNAcylation amino acid residues in EREG using NetOGlyc 4.0, indicating the potential for O-GlcNAcylation of EREG (**Figure 6C**). As expected, the level of O-GlcNAcylation in colon tissue was enhanced in AOM/DSS mice and was further strengthened by fructose supplementation (**Figure 6D**). Fructose supplementation in the culture medium of L929 fibroblasts also enhanced intracellular O-GlcNAcylation levels and the 30-kD form of EREG, exhibiting a time-dependent manner (**Figure 6E**). To further support that EREG can be O-GlcNAcylated by fructose, we performed a co-immunoprecipitation assay. We found that EREG interacts with O-GlcNAc transferase (OGT), and this interaction is stimulated by fructose, leading to enhanced O-GlcNAcylation of EREG (**Figure 6F**). Meanwhile, the O-GlcNAcylated EREG labeled by OGT were also increased in fructose-promoted CAC tumorigenesis (**Figure S10**). Another form of glycosylation, N-glycosylation, mediates the stabilization of EREG in HNSCC cells 34. We sought to investigate whether O-GlcNAcylation of EREG can maintain the stability. We observed that fructose stimulation enhanced the expression of EREG on the cell membrane, a response that was diminished by the O-GlcNAcylation inhibitor OSMI (**Figure 6G**). We sought to investigate whether O-GlcNAcylation of EREG can maintain the stability of EREG. We employed cycloheximide (CHX) to inhibit cellular protein synthesis and subsequently assessed the stability of EREG. We found that the degradation of EREG was diminished by fructose supplementation (**Figure 6H**).

To further characterize the role of fructose-induced O-GlcNAcylation of EREG on its stability and intercellular signal transduction, we constructed and expressed EREG plasmid in L929 cells. Following stimulation with fructose or OSMI-1, we co-incubated the culture supernatant with MC38 colon cancer cells for 24 hours to observe MC38 in cell proliferation and signaling pathways (**Figure 6I**). Supernatant of 2.5 mM fructose increased the viability of MC38, and this increase was attenuated by OSMI-1 (**Figure 6J**). Consistently, the supernatant of 5 mM and 10 mM fructose stimulation significantly enhanced the activation of EGFR and ERK in MC38 cells. The O-GlcNAcylation inhibitor OSMI-1 suppressed the fructose-induced activation (**Figure 6K**). The promotion of MC38 cell growth by O-GlcNAcylation of EREG was further confirmed by EdU assay (**Figure 6L and S11**). These data show that fructose-induced O-GlcNAcylation mediates O-GlcNAcylation of EREG promoted the activation of EGFR and ERK signaling in MC38 cells.

### Dieckol directly targets CNBP to alleviate colitis-associated colorectal cancer progression in inflammation-activated fibroblasts

**Figure 7:**
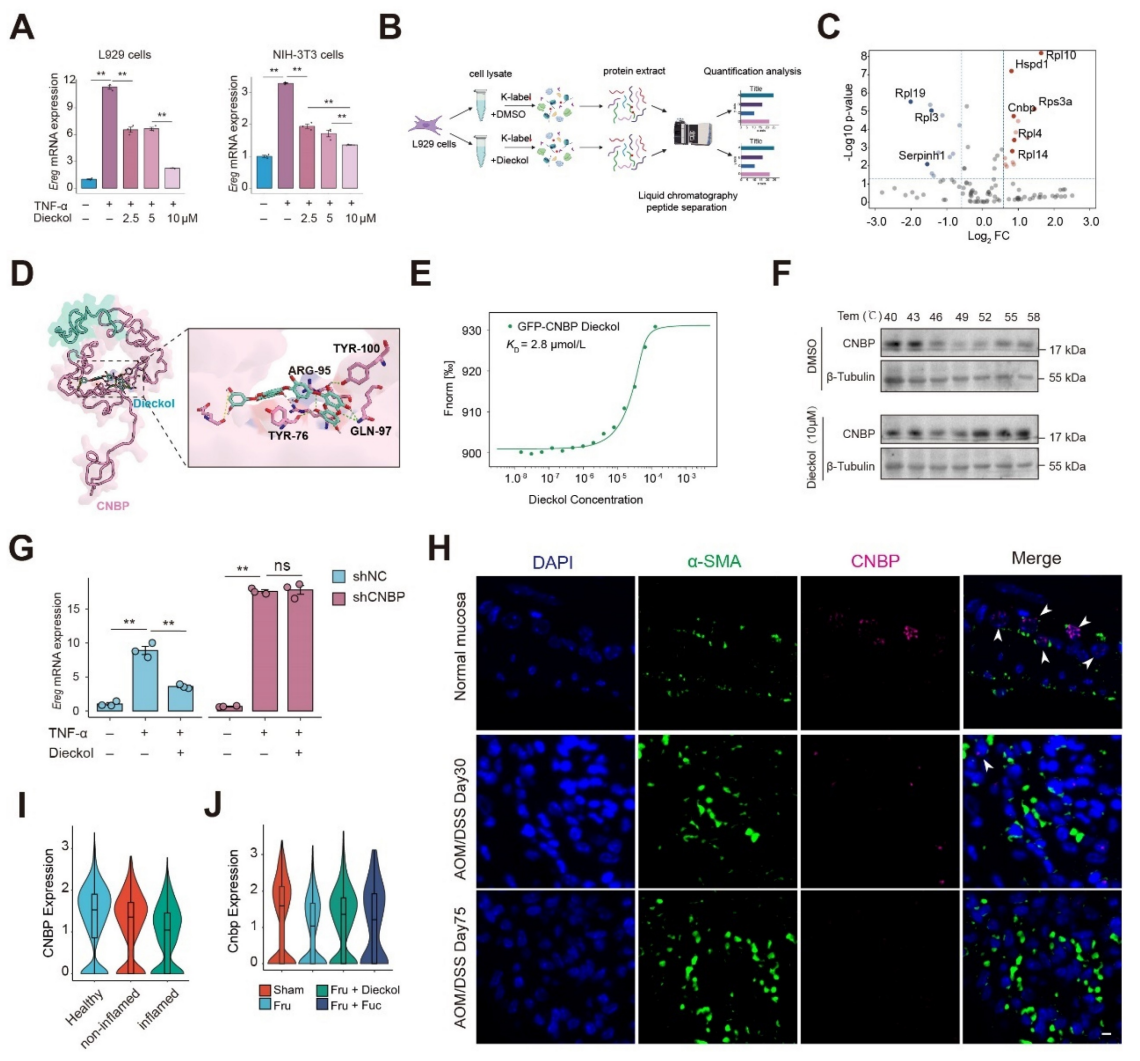
** Dieckol directly targets CNBP in inflammation-activated fibroblasts. A.** The expression of Ereg in response to TNF-α or different concentrations of dieckol stimulation in L929 or NIH-3T3 cells. **B.** Experimental workflow for TRAP assay. **C.** Dieckol directly binding proteins using TRAP assay. **D.** Docking analysis to predict the interaction sites of dieckol with CNBP. **E-F.** The binding of dieckol to CNBP were analyzed by MST **(E)** and CETSA **(F). G**. The mRNA expression of *Ereg* in NIH3T3 cells after lentiviral transduction under indicated stimulation.** H.** Immunofluorescence analysis of colocalization between CNBP, α-SMA, and DAPI in colon tissue. Scale bar = 10 μm. **I.** Violin plot showing CNBP expression in stromal cells across different clinical colon samples. Data were obtained from the publicly available single-cell RNA-seq dataset GSE231993. **J.** Violin plot showing CNBP expression in stromal cells across different groups.

The critical significance of drug targets in therapeutic development is undeniable. We next focused on elucidating the drug targets of kelp-derived compounds. Given that Dieckol has demonstrated superior therapeutic efficacy in CAC, we selected Dieckol as the compound for subsequent research (**Figure 4B-4C**). We first examined the effect of dieckol on *Ereg* expression under TNF-α stimulation. The data demonstrated that dieckol significantly attenuated *Ereg* expression in L929 fibroblasts and NIH-3T3 embryonic fibroblasts under inflammatory condition (**Figure 7A**). To further uncover the target of dieckol, we used the TRAP strategy to identify dieckol-binding proteins (**Figure 7B**). TRAP analysis revealed a pronounced increase in the intensity of the dieckol-bound protein, indicating that the accessibility of the probed region significantly increased due to steric repulsion induced by dieckol binding (**Figure 7C**). Given the high abundance of ribosomal protein family and heat shock protein family in cells, we hypothesize that CNBP is the binding protein of Dieckol. Subsequently, we predicted the binding between dieckol and CNBP according to the protein crystal structure of CNBP in the PDB library (**Figure 7D, Figure S12A**). Next, we used microscale thermophoresis (MST) to verify the binding between dieckol and CNBP. Results showed a higher binding affinity for dieckol and CNBP, the *K_D_* value was 2.8 μmol/L (**Figure 7E**). To verify the binding site between dieckol and CNBP, we transfected NIH-3T3 cells with mutant CNBP plasmids. The results showed that the Y76A, R95A, Q97A, and Y100A mutants exhibited binding affinities to dieckol that were several-fold lower than that of the wild-type CNBP plasmid. Notably, the Y100A mutation resulted in the most pronounced reduction in binding affinity, suggesting that Y100 may represent a critical residue for dieckol-CNBP interaction (**Figure S12B-E**). We confirmed the interaction between dieckol and CNBP using cell thermal shift assay (CETSA). The data showed that dieckol significantly enhanced the thermal stability of CNBP, even at higher temperatures, compared to the control group (DMSO treatment) (**Figure 7F**).

Next, we generated a CNBP knockdown NIH-3T3 cell line using lentiviral transduction. CNBP knockdown increased *Ereg* expression under inflammatory conditions and abolished the inhibitory effect of dieckol on EREG (**Figure 7G and Figure S12F**). We then examined CNBP expression in a mouse model of disease progression and observed that CNBP levels in inflammation-associated fibroblasts decreased over time (**Figure 7H**). We re-analyzed single-cell transcriptomic data from clinical colon samples, including four healthy individuals, four inflamed colonic regions from patients with colitis, and four non-inflamed regions from the same patients (**Figure S12G-H**). We examined CNBP expression in stromal cells and found that CNBP levels were markedly reduced in the inflamed colonic regions compared with both healthy controls and non-inflamed regions from colitis patients (**Figure 7I**). Further assessment of CNBP expression in αSMA-positive cells confirmed a marked decrease in CNBP levels (**Figure S12I-J**). Notably, intervention with dieckol and fucoidan stabilized CNBP expression, suggesting that these compounds preserve CNBP levels in the inflammatory microenvironment (**Figure 7J**).

### CNBP reins in EREG expression through promoter binding to restore homeostasis under inflammatory conditions

**Figure 8:**
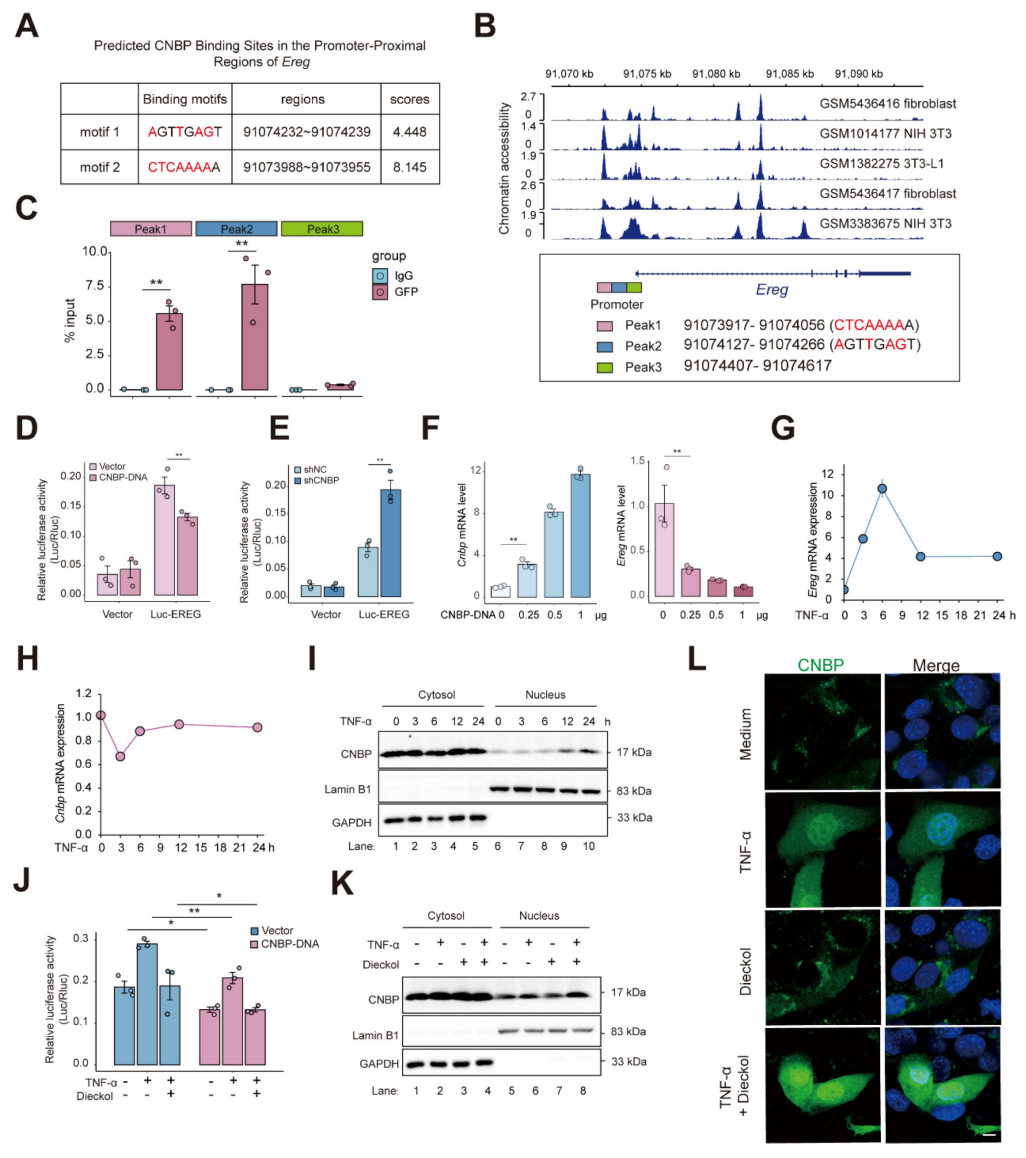
** CNBP represses *Ereg* transcription via promoter binding to maintain homeostasis under inflammatory conditions. A.** Schematic of the predicted CNBP binding sites within the *Ereg* promoter based on the CNBP binding motif by FIMO.** B.** Chromatin accessibility analysis of the Ereg locus showing that the predicted CNBP binding sites (https://db3.cistrome.org). **C.** ChIP-qPCR revealed the binding of CNBP to* Ereg* promoter or open chromatin regions in NIH-3T3 cells overexpressing GFP-EREG. **D-E.** Luciferase activity of *Ereg* in NIH-3T3 cells after 48-h transfection with plasmids **(D)** or lentiviral** (E)**.** F.** qPCR detection of mRNA expression of *Cnbp* and *Ereg* in overexpression CNBP in NIH-3T3 cells. **G-H.** Time-course of *Ereg* and *Cnbp* mRNA expression upon TNF stimulation. **I.** Subcellular fractionation and detection of cytoplasmic and nuclear proteins at various time points following TNF treatment. **J**. Luciferase activity of Ereg in NIH-3T3 cells after 48-h transfection with plasmids and subsequent treatment with dieckol. **K.** CNBP expression in cytoplasmic and nuclear fractions was analyzed following TNF and dieckol treatment. **L.** NIH-3T3 cells were transfected with GFP-CNBP plasmid, followed by TNF or dieckol stimulation for 12 hours after 48 hours of transfection. GFP localization was assessed by immunofluorescence. Scale bar = 5 μm.

Given the critical roles of CNBP as a broadly sequence-specific DNA- and RNA-binding protein involved in diverse cellular functions, we next examined whether CNBP might participate in the regulation of EREG 35. Based on the CNBP binding motif and the *Ereg* promoter sequence, we predicted potential CNBP binding sites within the *Ereg* promoter (**Figure 8A**) 36. We then examined chromatin accessibility of the *Ereg* locus and found that the predicted binding sites were located at the center of open chromatin peaks, suggesting that CNBP may regulate *Ereg* expression by binding to its promoter region (**Figure 8B**). Subsequently, we designed primers targeting the predicted CNBP binding sequences and performed ChIP-qPCR using a GFP-specific antibody on cells reconstituted with a GFP-CNBP plasmid. The results showed that CNBP specifically binds to the Peak1 and Peak2 regions of the *Ereg* promoter (**Figure 8C**). We also employed a reporter gene driven by the *Ereg* proximal promoter. Overexpression of CNBP via plasmid significantly suppressed the activity of the reporter gene, whereas knockdown of CNBP led to a notable increase in reporter activity (**Figure 8D-E**). Of note, EREG expression decreased in a dose-dependent manner with increasing amounts of transfected CNBP plasmid DNA (**Figure 8F**). Together, these results suggest that CNBP binds to the *Ereg* promoter and represses its transcription.

To further investigate the regulatory mechanism of CNBP on EREG under inflammatory conditions, we first examined the time-dependent expression of *Ereg* and *Cnbp* upon TNF stimulation, and monitored CNBP nuclear translocation. We found that *Ereg* expression peaked at 6 hours post-TNF treatment and subsequently stabilized over time, whereas *Cnbp* expression initially decreased upon TNF stimulation, then increased after 6 hours and reached a steady level (**Figure 8G-H**). Notably, CNBP translocated into the nucleus at 6 hours, with robust nuclear localization observed at 12 hours (**Figure 8I**). These findings suggest a feedback mechanism in which, in response to the rapid upregulation of EREG triggered by inflammatory signals, CNBP enters the nucleus to bind the *Ereg* promoter and repress its transcription, thereby contributing to the maintenance of cellular homeostasis.

Notably, under inflammatory conditions, dieckol enhanced the inhibitory effect of CNBP on *Ereg* promoter (**Figure 8J**). At the same time, dieckol promoting nuclear translocation of CNBP at 12 hours post-TNF stimulation, whereas dieckol alone did not induce CNBP nuclear translocation (**Figure 8K-L**). To functionally validate the requirement of CNBP in the dieckol-mediated regulatory axis, we performed CNBP knockdown and overexpression in NIH-3T3 cells, followed by TNF and/or dieckol treatment. Conditioned media were subsequently collected and applied to MC38 cells under a unified TNF background to exclude potential confounding cytotoxic effects. Conditioned media from dieckol-treated NIH-3T3 cells markedly suppressed MC38 proliferation. Notably, CNBP knockdown abolished the inhibitory effect of dieckol, as evidenced by an increased proportion of EdU-positive MC38 cells (**Figure S13A-C**). In contrast, conditioned media from CNBP-overexpressing cells significantly reduced MC38 proliferation, an effect that was further enhanced by dieckol treatment (**Figure S13D-F**). These results establish CNBP as an essential mediator of dieckol-induced tumor-suppressive signaling, functionally linking CNBP activity to the inhibition of tumor cell proliferation under inflammatory conditions.

Together, these findings suggest that dieckol reinforces CNBP-mediated repression of EREG in the context of inflammation, likely by facilitating CNBP's nuclear localization in response to inflammatory cues, thereby contributing to the maintenance of cellular homeostasis. Collectively, these findings indicate that CNBP reins in EREG expression to restore cellular homeostasis, and that dieckol can strengthen this regulatory pathway, highlighting a potential mechanism for modulating inflammation-driven *Ereg* expression.

## Discussion

While previous studies have highlighted the tumor-promoting role of high fructose intake on sporadic colon cancer and acute colitis, systematic research on colitis-associated colorectal cancer and effective treatment strategies remain scarce 37-38. In this study, we demonstrated that high fructose significantly exacerbates intestinal inflammation during the colitis stage in mice, further promoting tumor growth and progression. Current research on the impact of fructose on tissue homeostasis predominantly centers on its modulation of gut microbiota and metabolic pathways, which subsequently mediate systemic equilibrium 39,40. Additionally, another significant report demonstrated that fructose intake enhances the survival rate of intestinal villus cells, leading to longer intestinal villi that absorb more nutrients 41. These findings align with our research, which indicates that high fructose intake results in damage to the intestinal mucosal barrier, increased inflammation, and enhanced malignant proliferation of epithelial cells.

More importantly, as colorectal cancer is an epithelial tumor, our single-cell transcriptomic analysis revealed that fructose intake enhances the interactions between epithelial and stromal cells. It is well known that cytokines and growth factors secreted by stromal cells activate the tumorigenic potential of epithelial cells 42. We identified receptor-ligand pairs that are significantly enriched during fructose-accelerated CAC development. Targeting these pairs offers new opportunities for drug discovery and therapeutic strategies for colorectal cancer. Interestingly, we incorporated a library of medicine-food homologous into our study. These substances are well-known for their edibility and high pharmacological activity. We selected them to address the long-term relationship between the development of CAC and daily fructose intake. Our goal was to identify a treatment strategy or preventive measure that could be safely administered over an extended period. The drugCIPHER, based on network pharmacology, assesses spatial similarity (i.e., pharmacological similarity) and structural similarity of drugs to rank receptor-ligands across the entire genome according to network proximity 17. Through this screening, we identified kelp as a promising medicinal and edible substance. Furthermore, our screening process revealed that kelp primarily intervenes in the EREG-EGFR pathway. The secretion of EREG from tumor-associated fibroblasts can promote the proliferation of intestinal epithelial cells via the ERK pathway, thereby driving the growth of colitis-associated tumors 30. Previous studies have demonstrated that EREG can induce EGFR phosphorylation in colonic epithelial cells, leading to ERK activation and subsequent cell proliferation 43. The EREG/ERK pathway is particularly relevant to inflammation-induced colorectal tumors, as mucosal inflammation significantly increases the production of effective EREG inducers, such as LPS and TNF-α 44. Subsequently, EREG may further enhance its own production through an auto-regulatory feedback loop in tumor-associated fibroblasts, explaining the specific relevance of EREG to inflammation-related tumors. The binding of different ligands determines the fate of EGFR. Compared to other ligands, EREG exhibits a pronounced tendency to recycle EGFR back to the plasma membrane, thereby inducing more sustained EGFR signaling 44,45. This further suggests the important role of EREG in inflammation related diseases or tumors.

In our study, we confirmed that EREG is expressed by inflammatory fibroblasts in the high-fructose promoted AOM/DSS model, with its secretion increasing alongside tumor development and fructose intake. We propose that fructose stabilizes localization of EREG on the cell membrane and its paracrine secretion by O-GlcNAcylation. This increased stability partially enhances the sustained activation of the EGFR pathway in epithelial cells. These findings suggest two potential mechanisms by which dietary fructose promotes colitis-associated colorectal cancer: on the one hand, fructose exacerbates intestinal inflammation, thereby stimulating elevated EREG transcription; on the other hand, fructose enhances EREG stability through O-GlcNAcylation modification, leading to persistent activation of the EGFR-ERK signaling cascade.

Finally, we selected dieckol as the molecular target for our research, given that kelp-derived phlorotannins have been approved by the European Union as novel foods and dietary supplements. Our findings indicate that dieckol targets the RNA/DNA-binding protein CNBP, disrupting its interaction with the EREG promoter, thereby attenuating the progression of colitis-associated colorectal cancer. Our findings establish CNBP as a pivotal negative regulator of EREG expression under inflammatory conditions, acting through direct binding to the *Ereg* promoter. The dynamic feedback mechanism whereby CNBP responds to rapid EREG upregulation to restrain excessive transcription and maintain cellular homeostasis. These findings not only reveal a previously unrecognized mechanism controlling EREG expression under inflammatory conditions, but also have important implications in chronic inflammatory diseases, where sustained EREG upregulation may promote pathological proliferation and tissue damage. By highlighting CNBP as a negative regulator that maintains EREG homeostasis, we identify a potential regulatory node that could be targeted to intervene in chronic inflammation-associated disorders, such as chronic colitis, endometrial hyperplasia, or other inflammation-driven tissue pathologies. Notably, this regulatory mechanism is fundamentally distinct from previously reported functions of CNBP, thereby revealing an unanticipated layer of control in inflammatory contexts 18-20, 46. Importantly, we demonstrate that dieckol reinforces this regulatory axis by promoting CNBP nuclear localization in the presence of inflammatory stimuli, thereby enhancing repression of EREG. More broadly, our results highlight how transcriptional repressors like CNBP can act as homeostatic brakes to fine-tune the expression of inflammation-associated genes, and suggest that pharmacological modulation of such regulators may represent a promising strategy to mitigate inflammation-driven pathologies.

## Conclusions

This study uncovers a multi-omics-defined mechanism by which dietary fructose exacerbates colitis-associated colorectal cancer through EREG-EGFR activation and highlights CNBP as a transcriptional brake on *Ereg* expression. Our findings emphasize the value of integrating food-based and systems-level approaches in chronic disease prevention and support the development of Laminaria japonica-derived compounds as safe, long-term dietary agents for managing colitis-associated colorectal cancer.

## Supplementary Material

Supplementary methods, figures and tables.

## Data Availability

The datasets generated and/or analysed during the current study are available in the NCBI BioProject repository under accession number PRJNA1268991.
