# Supplementary material for: Dietary targeting of CNBP to rein in the EREG-EGFR cascade and restore homeostasis in colitis-associated colorectal cancer
Source: Int J Biol Sci. 2026 Feb 26;22(6):3204–23. doi: 10.7150/ijbs.128354 (PMC13050469; doi:10.7150/ijbs.128354)
Supplement: Supplementary file 1 — Supplementary methods, figures and tables. [file ijbsv22p3204s1.pdf]

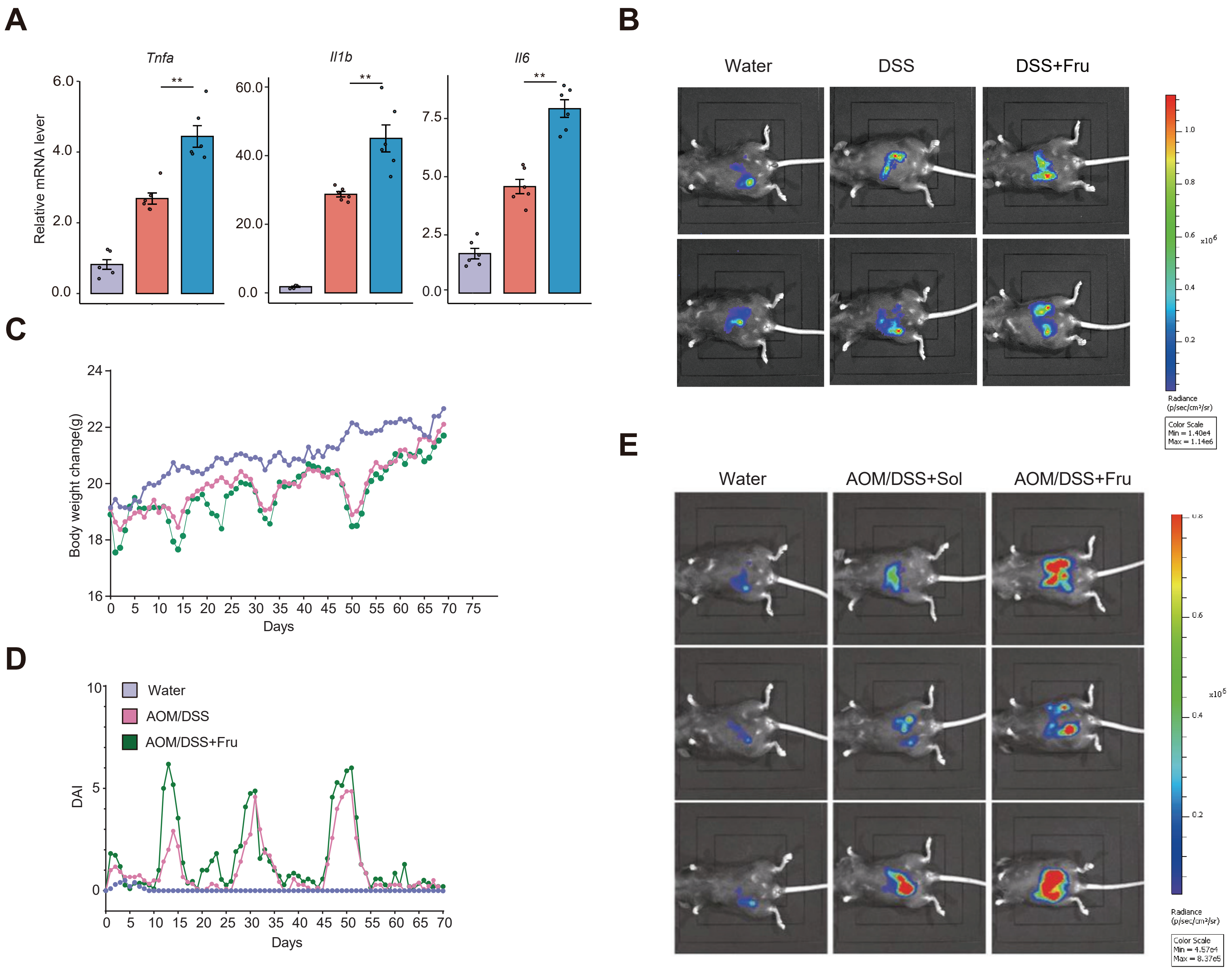

A
Tnfa
Il1b
Il6
Relative mRNA lever
6.0
4.0
2.0
0.0
60.0
40.0
20.0
0.0
7.5
5.0
2.5
0.0
**
B
Water
DSS
DSS+Fru
Radiance (p/sec/cm²/sr)
Color Scale Min = 1.40e4 Max = 1.14e6
C
Body weight change(g)
Days
D
DAI
Water
AOM/DSS
AOM/DSS+Fru
Days
E
Water
AOM/DSS+Sol
AOM/DSS+Fru
Radiance (p/sec/cm²/sr)
Color Scale Min = 4.57e4 Max = 8.37e5

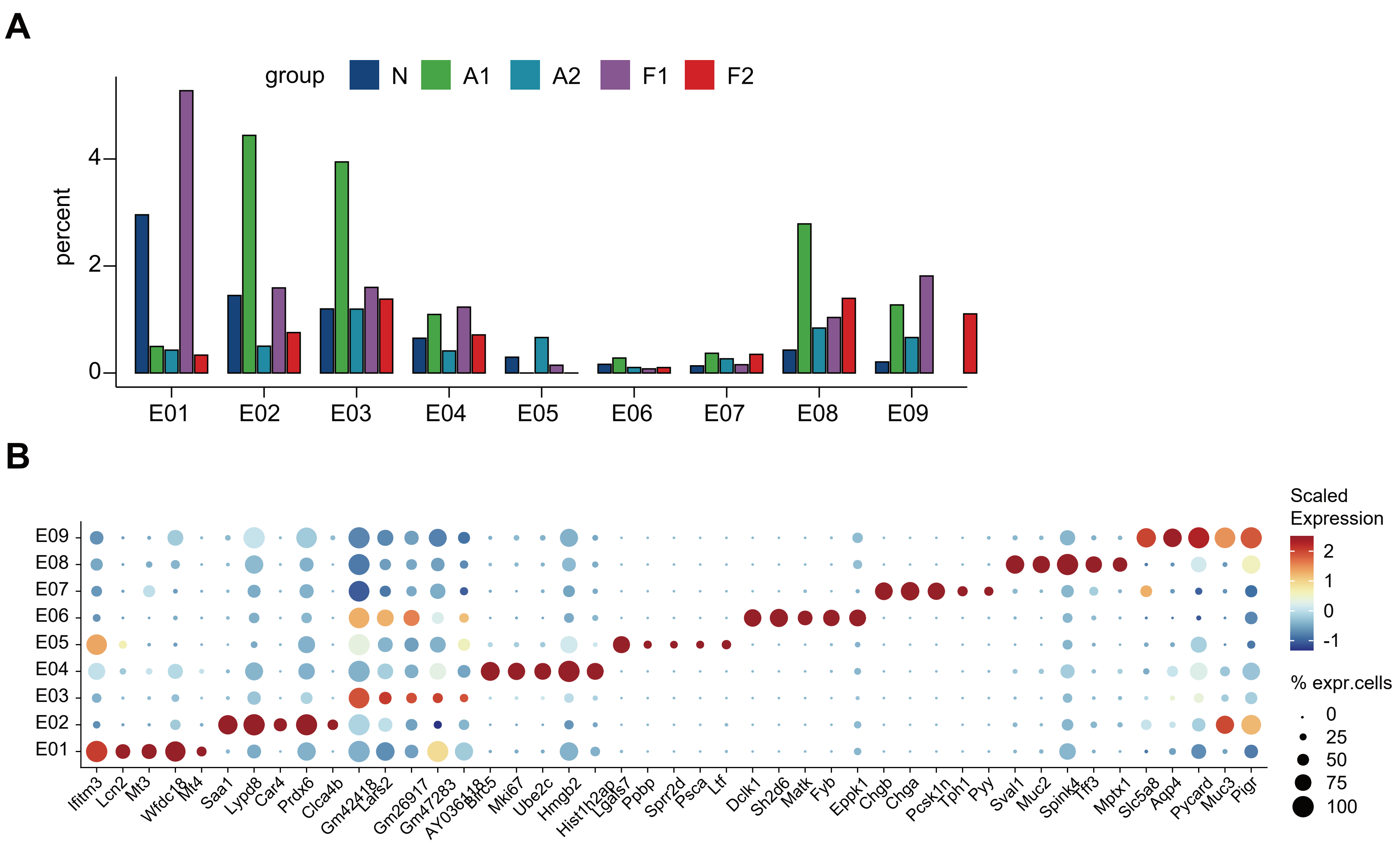

A
group
N
A1
A2
F1
F2
percent
0
2
4
E01
E02
E03
E04
E05
E06
E07
E08
E09
B
Scaled Expression
2
1
0
-1
% expr.cells
0
25
50
75
100
E09
E08
E07
E06
E05
E04
E03
E02
E01
Ifitm3
Lcn2
Mt3
Wfdc18
Mt4
Saa1
Lypd8
Car4
Prdx6
Clca4b
Gm42418
Lars2
Gm26917
Gm47283
Birc5
Mki67
Ube2c
Hmgb2
Lgals7
Ppbp
Sprr2d
Psca
Ltf
Dclk1
Sh2d6
Matk
Fyb
Eppk1
Chgb
Chga
Pcsk1n
Tph1
Pyy
Sval1
Muc2
Spink4
Tff3
Mptx1
Slc5a8
Aqp4
Pycard
Muc3
Pigr

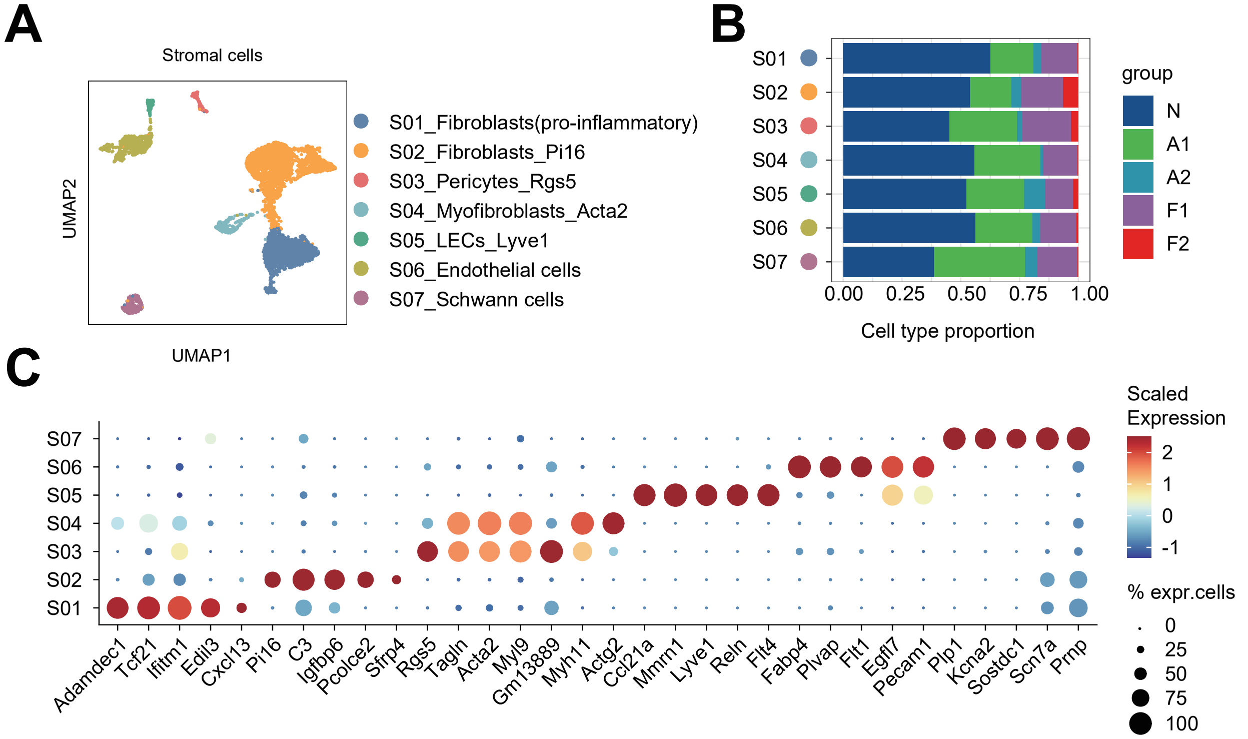
A
Stromal cells
UMAP2
UMAP1
S01_Fibroblasts(pro-inflammatory)
S02_Fibroblasts_Pi16
S03_Pericytes_Rgs5
S04_Myofibroblasts_Acta2
S05_LECs_Lyve1
S06_Endothelial cells
S07_Schwann cells
B
S01
S02
S03
S04
S05
S06
S07
0.00
0.25
0.50
0.75
1.00
Cell type proportion
group
N
A1
A2
F1
F2
C
S07
S06
S05
S04
S03
S02
S01
Adamdec1
Tcf21
Ifitm1
Edil3
Cxcl13
Pi16
C3
Igfbp6
Pcolce2
Sfrp4
Rgs5
Tagln
Acta2
Myl9
Gm13889
Myh11
Actg2
Ccl21a
Mmrn1
Lyve1
Reln
Flt4
Fabp4
Plvap
Flt1
Egfl7
Pecam1
Plp1
Kcna2
Sostdc1
Scn7a
Prnp
Scaled
Expression
2
1
0
-1
% expr.cells
0
25
50
75
100

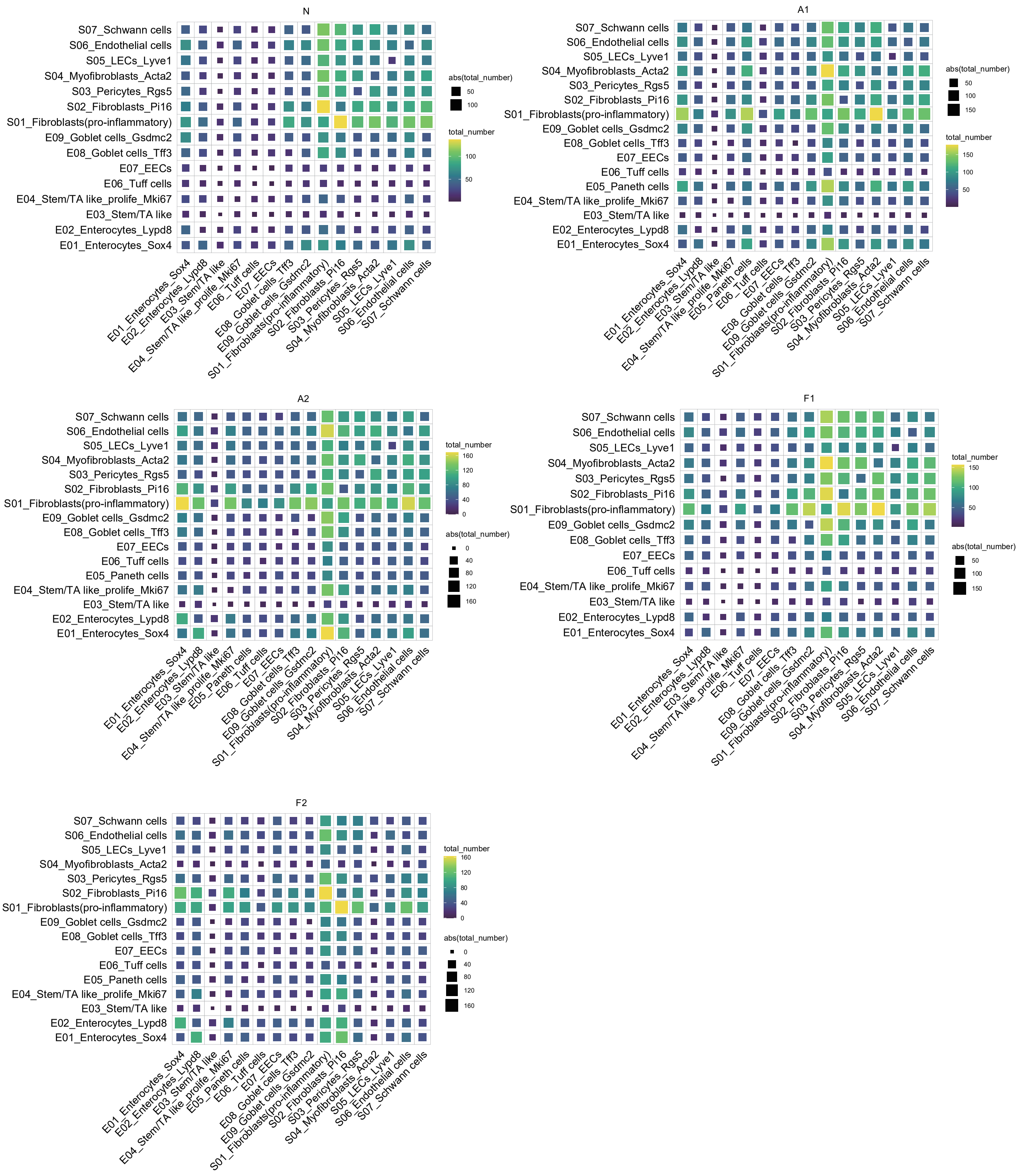
N
A1
A2
F1
F2
total_number
abs(total_number)
S07_Schwann cells
S06_Endothelial cells
S05_LECs_Lyve1
S04_Myofibroblasts_Acta2
S03_Pericytes_Rgs5
S02_Fibroblasts_Pi16
S01_Fibroblasts(pro-inflammatory)
E09_Goblet cells_Gsdmc2
E08_Goblet cells_Tff3
E07_EECs
E06_Tuff cells
E05_Paneth cells
E04_Stem/TA like_prolife_Mki67
E03_Stem/TA like
E02_Enterocytes_Lypd8
E01_Enterocytes_Sox4

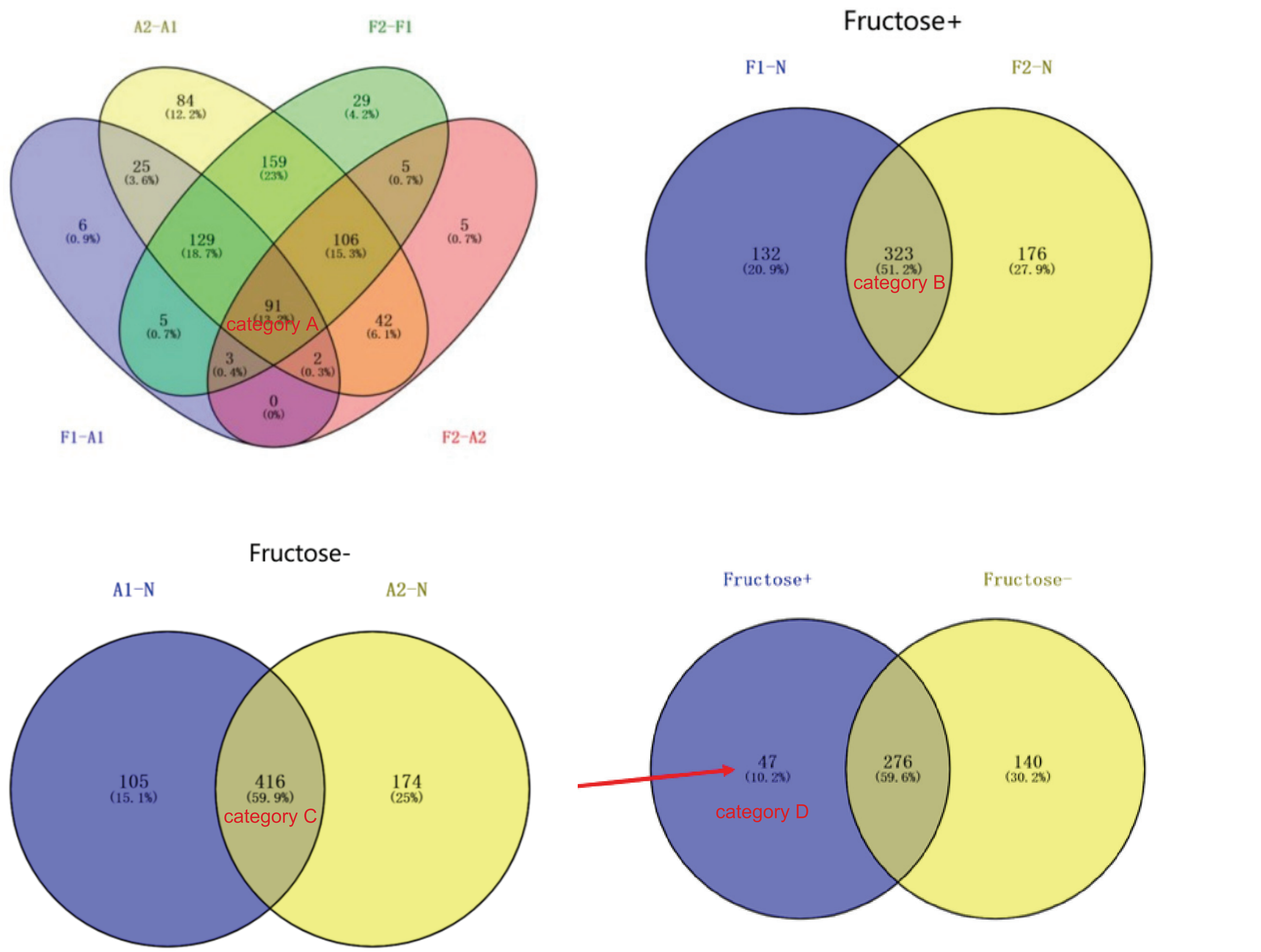
A2-A1
F2-F1
F1-A1
F2-A2
84 (12.2%)
29 (4.2%)
25 (3.6%)
159 (23%)
5 (0.7%)
6 (0.9%)
129 (18.7%)
106 (15.3%)
5 (0.7%)
91
category A
5 (0.7%)
42 (6.1%)
3 (0.4%)
2 (0.3%)
0 (0%)
Fructose+
F1-N
F2-N
132 (20.9%)
323 (51.2%)
category B
176 (27.9%)
Fructose-
A1-N
A2-N
105 (15.1%)
416 (59.9%)
category C
174 (25%)
Fructose+
Fructose-
47 (10.2%)
category D
276 (59.6%)
140 (30.2%)

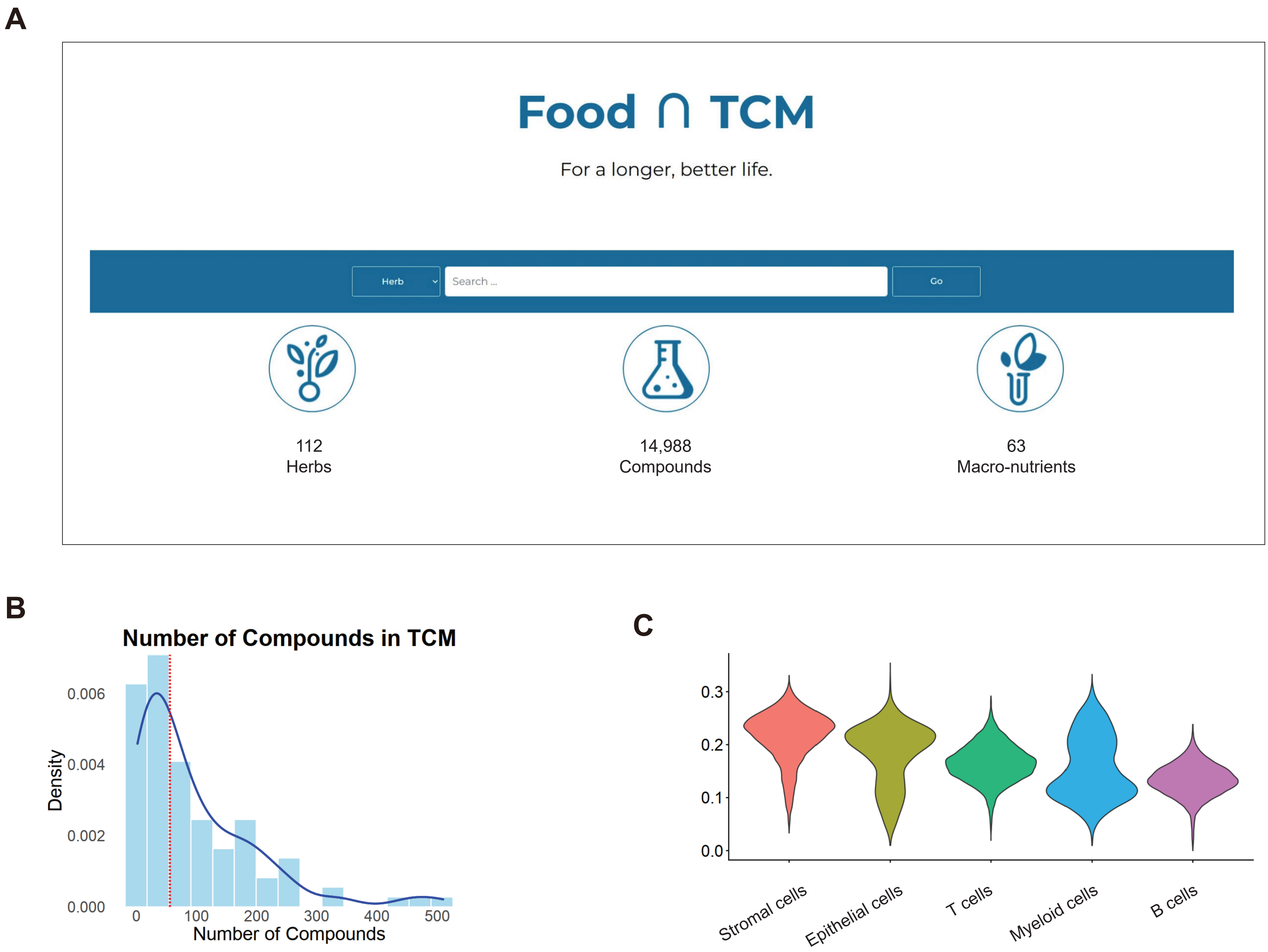
A
Food ∩ TCM
For a longer, better life.
Herb
Search ...
Go
112
Herbs
14,988
Compounds
63
Macro-nutrients
B
Number of Compounds in TCM
Density
0.006
0.004
0.002
0.000
0
100
200
300
400
500
Number of Compounds
C
0.3
0.2
0.1
0.0
Stromal cells
Epithelial cells
T cells
Myeloid cells
B cells

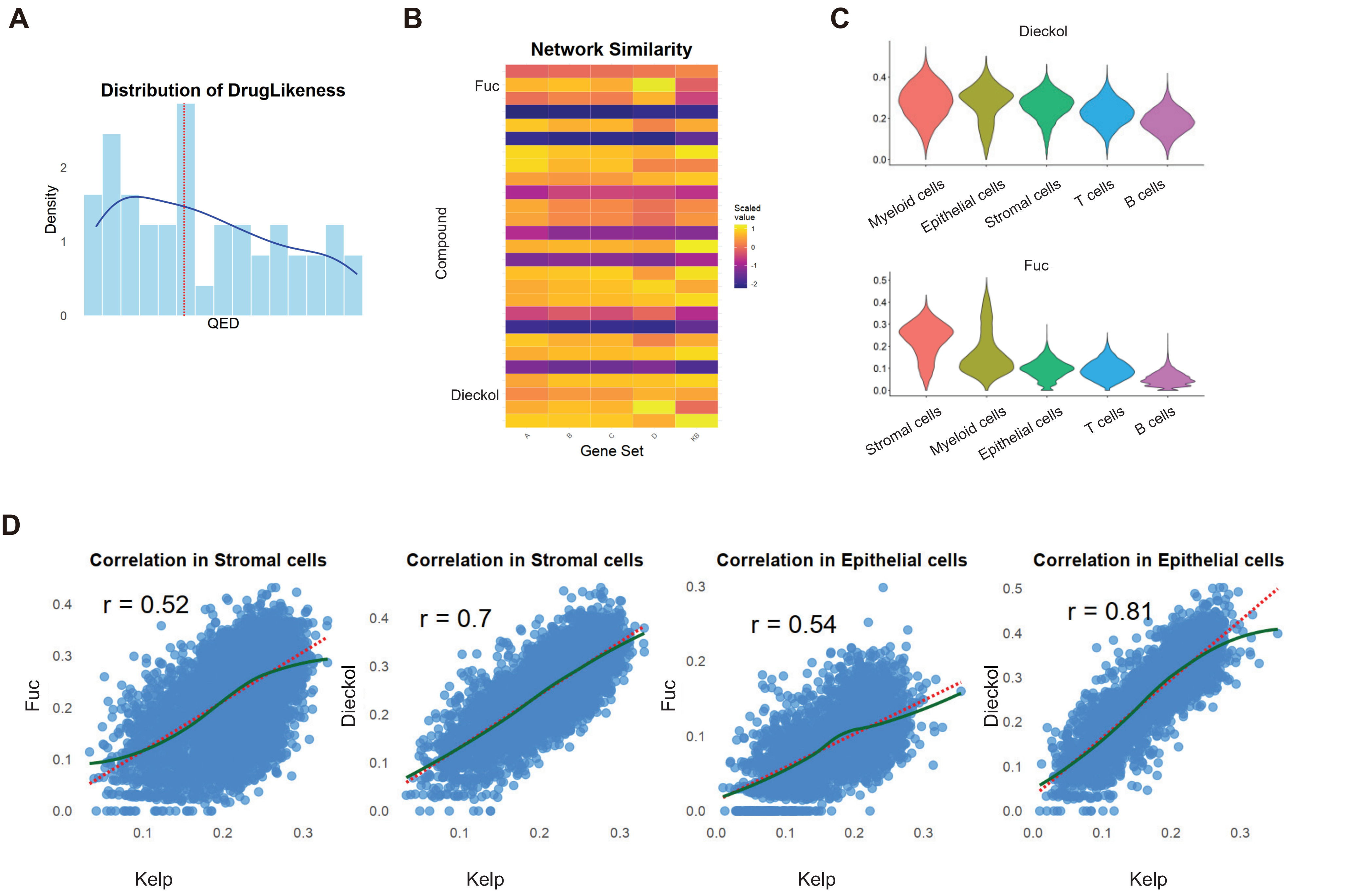
A
Distribution of DrugLikeness
Density
QED
B
Network Similarity
Fuc
Compound
Dieckol
Gene Set
Scaled value
C
Dieckol
Myeloid cells
Epithelial cells
Stromal cells
T cells
B cells
Fuc
Stromal cells
Myeloid cells
Epithelial cells
T cells
B cells
D
Correlation in Stromal cells
r = 0.52
Fuc
Kelp
Correlation in Stromal cells
r = 0.7
Dieckol
Kelp
Correlation in Epithelial cells
r = 0.54
Fuc
Kelp
Correlation in Epithelial cells
r = 0.81
Dieckol
Kelp

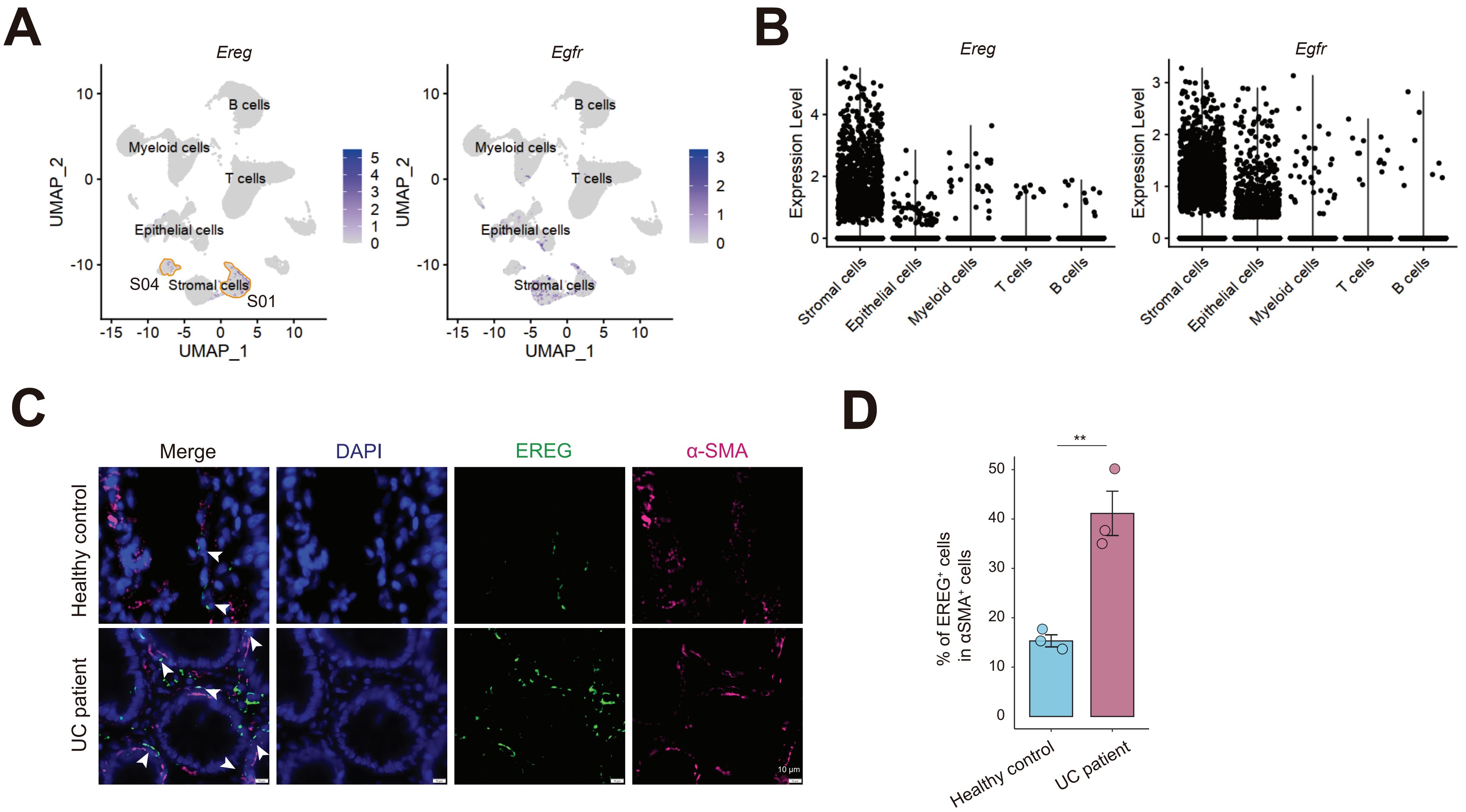
A
Ereg
Egfr
B cells
Myeloid cells
T cells
Epithelial cells
Stromal cells
S04
S01
UMAP_1
UMAP_2
B
Expression Level
Stromal cells
Epithelial cells
Myeloid cells
T cells
B cells
C
Merge
DAPI
EREG
α-SMA
Healthy control
UC patient
10 μm
D
% of EREG⁺ cells in αSMA⁺ cells
**
Healthy control
UC patient

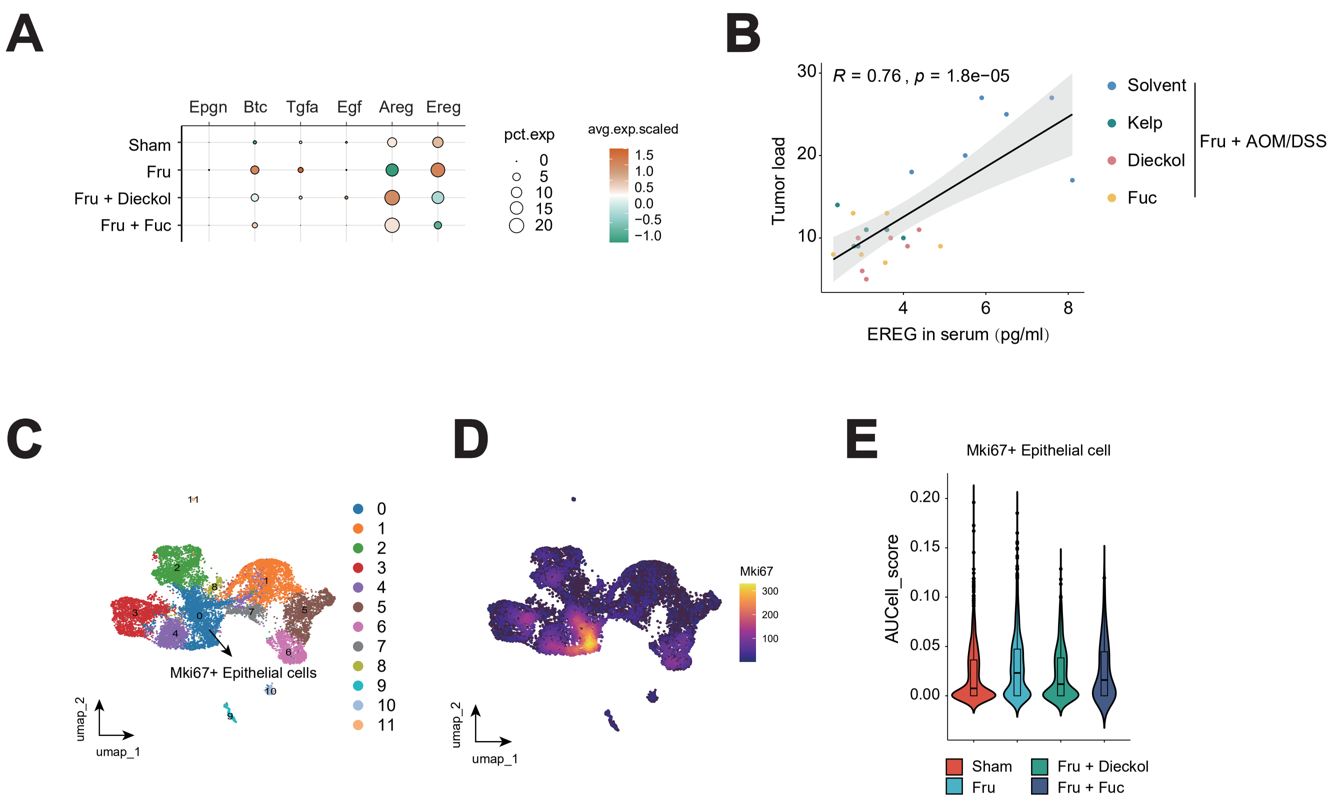
A
Epgn
Btc
Tgfa
Egf
Areg
Ereg
Sham
Fru
Fru + Dieckol
Fru + Fuc
pct.exp
0
5
10
15
20
avg.exp.scaled
1.5
1.0
0.5
0.0
−0.5
−1.0
B
R = 0.76 , p = 1.8e−05
Tumor load
30
20
10
4
6
8
EREG in serum (pg/ml)
Solvent
Kelp
Dieckol
Fuc
Fru + AOM/DSS
C
0
1
2
3
4
5
6
7
8
9
10
11
Mki67+ Epithelial cells
umap_2
umap_1
D
Mki67
300
200
100
umap_2
umap_1
E
Mki67+ Epithelial cell
AUCell_score
0.20
0.15
0.10
0.05
0.00
Sham
Fru
Fru + Dieckol
Fru + Fuc

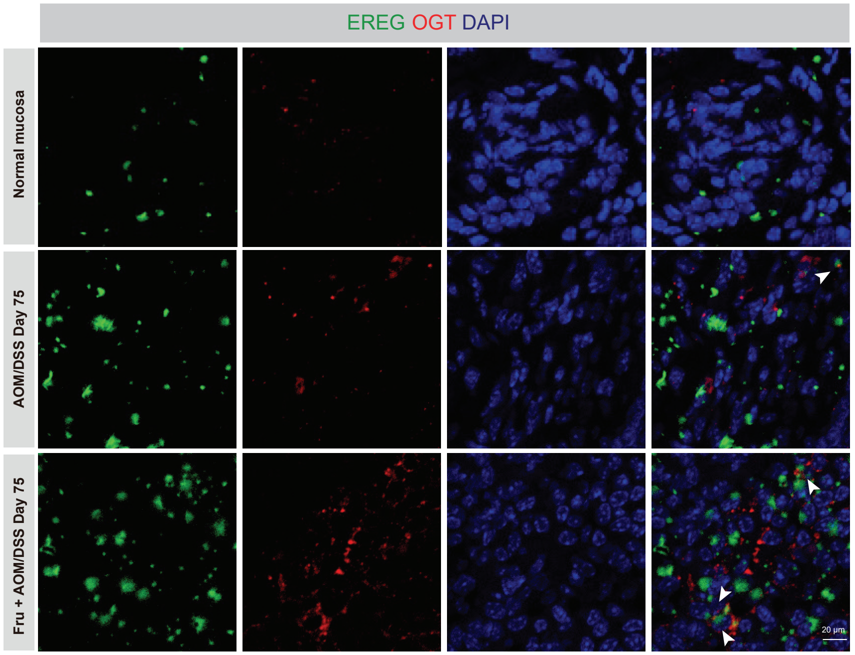
EREG OGT DAPI
Normal mucosa
AOM/DSS Day 75
Fru + AOM/DSS Day 75
20 μm

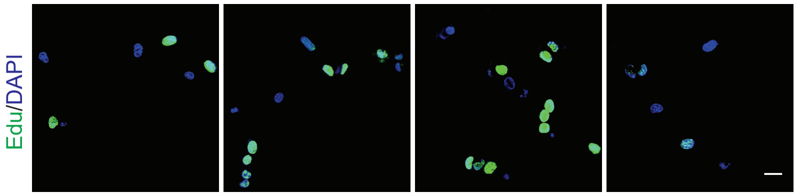

EREG plasmid	−	+	+	+
Fru	−	−	10	10
OSMI-1	−	−	−	+

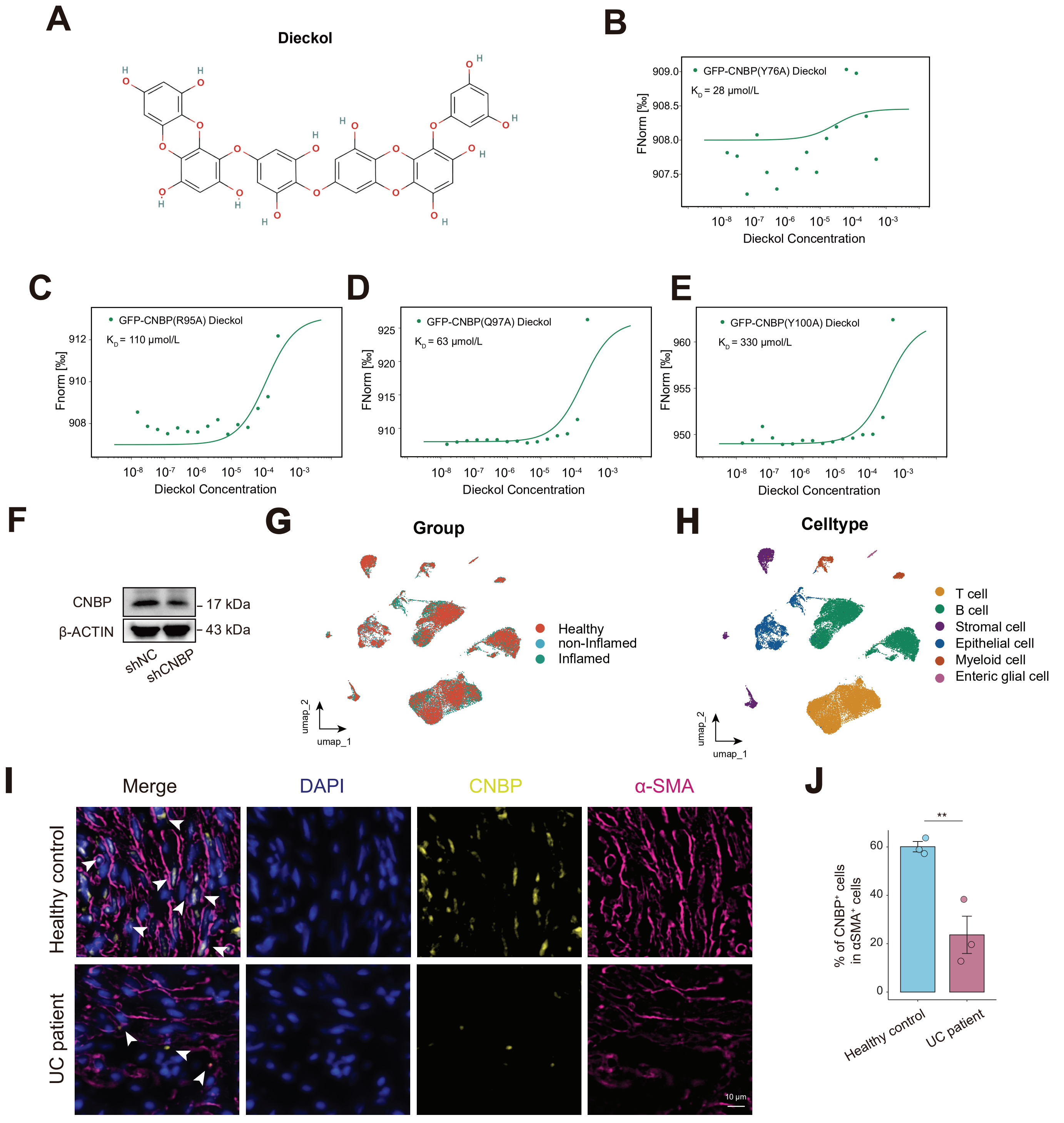
A
Dieckol
B
GFP-CNBP(Y76A) Dieckol
K_D = 28 μmol/L
FNorm [‰]
Dieckol Concentration
C
GFP-CNBP(R95A) Dieckol
K_D = 110 μmol/L
Fnorm [‰]
Dieckol Concentration
D
GFP-CNBP(Q97A) Dieckol
K_D = 63 μmol/L
FNorm [‰]
Dieckol Concentration
E
GFP-CNBP(Y100A) Dieckol
K_D = 330 μmol/L
FNorm [‰]
Dieckol Concentration
F
CNBP
β-ACTIN
17 kDa
43 kDa
shNC
shCNBP
G
Group
Healthy
non-Inflamed
Inflamed
umap_2
umap_1
H
Celltype
T cell
B cell
Stromal cell
Epithelial cell
Myeloid cell
Enteric glial cell
umap_2
umap_1
I
Merge
DAPI
CNBP
α-SMA
Healthy control
UC patient
10 μm
J
% of CNBP+ cells in αSMA+ cells
Healthy control
UC patient
**

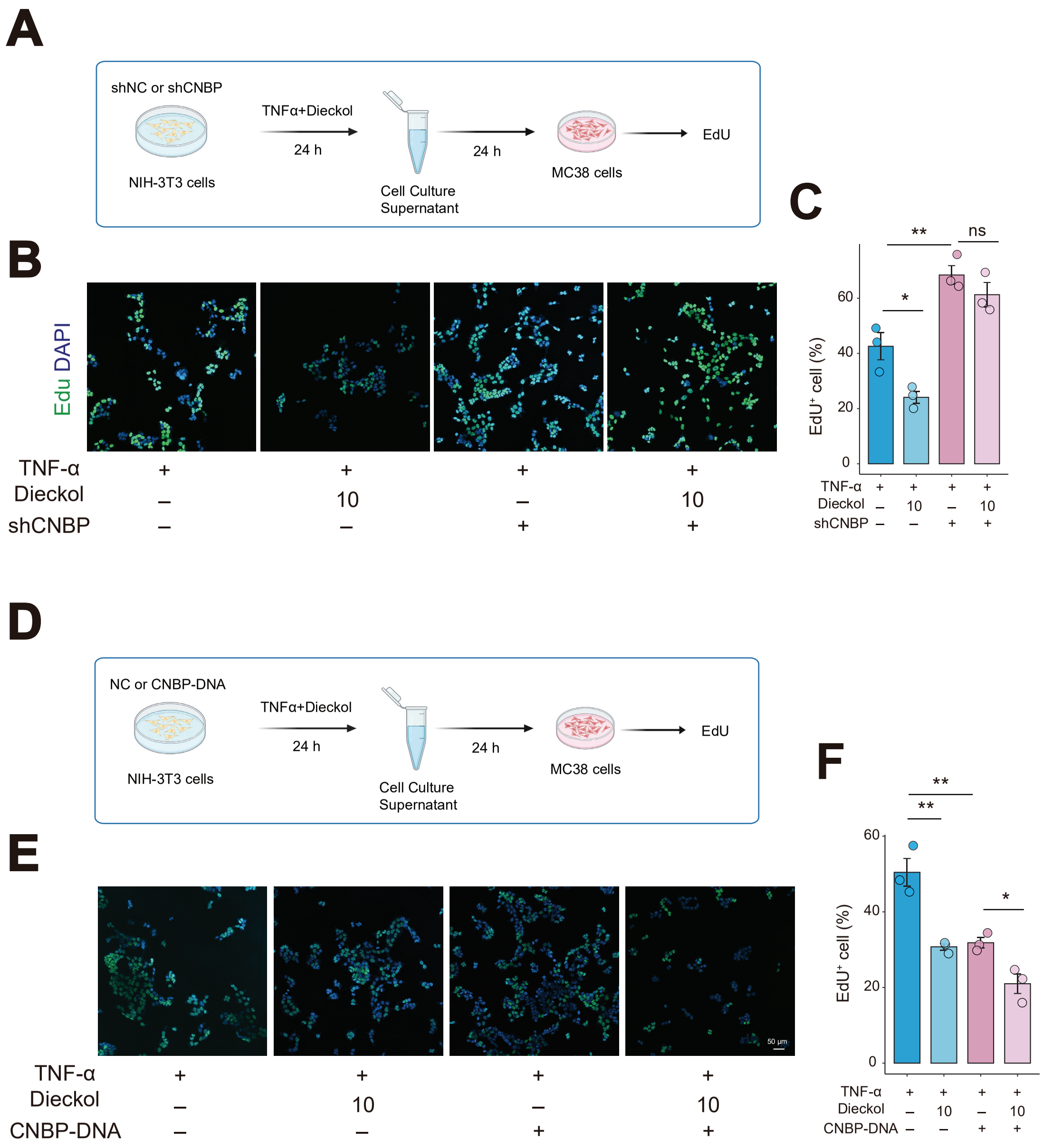
A
shNC or shCNBP
NIH-3T3 cells
TNFα+Dieckol
24 h
Cell Culture Supernatant
24 h
MC38 cells
EdU
B
Edu DAPI
TNF-α + + + +
Dieckol – 10 – 10
shCNBP – – + +
C
EdU⁺ cell (%)
0
20
40
60
**
ns
*
TNF-α + + + +
Dieckol – 10 – 10
shCNBP – – + +
D
NC or CNBP-DNA
NIH-3T3 cells
TNFα+Dieckol
24 h
Cell Culture Supernatant
24 h
MC38 cells
EdU
E
50 μm
TNF-α + + + +
Dieckol – 10 – 10
CNBP-DNA – – + +
F
EdU⁺ cell (%)
0
20
40
60
**
**
*
TNF-α + + + +
Dieckol – 10 – 10
CNBP-DNA – – + +

Supplementary Table 1. Antibodies used in the study

Antibodies	Source	Identifier
EREG	Santa Cruz	sc-376284
p-ERK	CST	4370
ERK	CST	4695
p-EGFR	CST	11862
EGFR	Santa Cruz	sc-81488
OGT	Santa Cruz	sc-74546
O-GlcNAc	Abcam	ab2739
Ki67	Abcam	ab15580
HA-Tag	CST	3724
α-SMA	CST	19245
GAPDH	Affinity	T0004
β-ACTIN	Affinity	AF7018
β-Tublin	Affinity	T0023
GFP-Tag	Proteintech	50430-2-AP
CNBP	Santa Cruz	sc-515387

Supplementary Table 2. Compound information

Name	Source	Identifier
DSS	MP Biomedicals	0216011090
D- (−)-fructose	Sigma	F0127
AOM	Sigma	A5486
Fucoidan	Yuanye	S11142
Dieckol	MCE	HY-147059
CCK-8	MCE	HY-K0301
EdU	Beyotime	C0071S
HiScript III RT SuperMix for qPCR	Vazyme	R323-01
SYBR Green Mix	Vazyme	Q712-02
Trizol	Takara	9109
L-012	Wako	120-04891
DAPI	Thermo Fisher Scientific	D3751
Alexa Fluor™594	Thermo Fisher Scientific	A11005
Alexa Fluor™488	Thermo Fisher Scientific	A11008
Multiple immunofluorescence kit	Shang Hai GuduoBio Technology Co. Ltd	HYDS0045
IHC Detect Kit	Proteintech Group Inc	PK10006
Protein A/G	Thermo Fisher Scientific	88802
ChIP assay kit	Beyotime	P2078
Dual Luciferase Reporter Gene Assay Kit	Beyotime	RG027

Mouse EREG(Epiregulin) ELISA Kit	ELK Biotechnology	ELK4009
TNF-α	Peprotech	PMC3016
OSMI-1	MCE	HY-119738
FITC-Dextran	MCE	HY-128868G
Lipofectamine™ 3000	Thermo Fisher Scientific	L3000015
Cycloheximide	MCE	HY-12320

Supplementary Table 3. Oligonucleotides used in the study information

qPCR Primers	Sequence
Mouse-*Ereg*	5'-ACGGGGATCGTCTTCCATCT-3'
	5'-CGCTGCTTGTGTAGGTTCCC-3'
Mouse- *Ereg* promoter (ChIP)	5'-AAGGCATGCTGAAGGGACTC-3'
	5'-GATTCCAGTGCCGAACCAGA-3'
Mouse- *Ereg* Peak1 (ChIP)	5'-CGACAGCACTCAGACTCTCC-3'
	5'-CCATGAGTCAGGACCCAACC-3'
Mouse- *Ereg* Peak2 (ChIP)	5'-GGATCAGCTCGGTTCCAACT-3'
	5'-AAAGCAGCGTCAAGACCCAA-3'
Mouse-*Tnfa*	5'-ATGAGCACAGAAAGCATGATC-3'
	5'-TACAGGCTTGTCACTCGAATT-3'
Mouse-*Il1b*	5'-GAAATGCCACCTTTTGACAGTG-3'
	5'-TGGATGCTCTCATCAGGACAG-3'
Mouse- *Il6*	5'-TAGTCCTTCCTACCCCAATTTCC-3'
	5'-TTGGTCCTTAGCCACTCCTTC-3'

Methods

High-fructose exacerbated DSS-induced colitis model and colitis-associated cancer (CAC) model

Eight-week-old male C57BL/6 mice were obtained from GemPharmatech Co. Ltd. and housed under controlled conditions (21±3°C, 12-hour light/dark cycle). All experiments were approved by an Institutional Animal Care and Use Committee (IACUC) at the Model Animal Research Center of Nanjing University (IACUC2506005). For the high-fructose exacerbated colitis model, thirty 8-week-old

male C57BL/6 mice were randomly divided into three groups. The Fructose+DSS group received drinking water supplemented with 30% fructose, which was refreshed every 48 hours for 4 consecutive weeks. The remaining groups consumed standard drinking water. At week 5, 2.5% DSS was administered in the drinking water of both DSS and Fructose+DSS groups, with the latter maintaining 30% fructose supplementation. On day 6 post-DSS administration, the DSS solution was replaced with either standard water or 30% fructose water. Body weight, fecal consistency, and anal hygiene status were monitored throughout the experiment. All mice were euthanized on day 8 post-DSS initiation following a 12-hour fasting period.

For the CAC model, the mice received a single intraperitoneal injection of 7.5 mg/kg AOM solution on day 0. The AOM/DSS+Fructose group was provided with 30% fructose-supplemented drinking water continuously throughout the entire modeling period. On day 5, both AOM/DSS and AOM/DSS+Fructose groups were administered 2.5% DSS in drinking water for 5 consecutive days. This was followed by a 14-day recovery period starting, during which mice received either standard water or 30% fructose water. The DSS challenge-recovery cycle was repeated twice. Body weight, fecal consistency, and anal health were monitored throughout the experiment. Tissues were harvested for analysis at the inflammatory phase (tumor initiation, day 30) and adenoma phase (day 75). At the experimental endpoint, colons were opened longitudinally and visible tumors (≥1 mm in diameter) were counted. Tumor load was determined by summing the calculated areas of all identified tumors for each mouse. Tumor length and width were measured using an electronic caliper, and tumor area was calculated accordingly. All measurements were performed in a blinded manner.

To evaluate the ameliorative effects of the selected medicinal-food homologous compounds on high-fructose diet-aggravated colitis-associated colorectal cancer, mice were administered daily via intragastric gavage with Kelp extract (200 mg/kg), dieckol (100 mg/kg), and fucoidan (100 mg/kg). According to previous reports, the disease activity index (DAI) was determined based on three parameters: weight loss, stool consistency, and the severity of intestinal bleeding.

Kelp extract

First, thoroughly wash dried Kelp (Laminaria japonica) with abundant distilled water to remove surface dust, salts, and soluble impurities. Kelp fragments were mixed with distilled water, followed by the addition of enzymatic agents (pectinase and cellulase). The suspension was stirred at 50°C for 24 hours, subsequently centrifuged at 3000 g for 20 minutes at 4°C, and vacuum-filtered. Three volumes of 60% ethanol were then added for precipitation. After 18 hours of extraction, the solution was filtered, concentrated using a rotary evaporator, and finally spray-dried to obtain the powdered extract [21].

Cell culture

L929 cells were cultured in DMEM (11965092, Gibco) supplemented with 10% fetal

bovine serum (10100147, Gibco) and 1% penicillin-streptomycin (C0222, Beyotime); NIH-3T3 cells were maintained in DMEM containing 10% newborn calf serum (26010074, Gibco). Both cell lines were incubated at 37°C with 5% CO2.

Single-cell RNA sequencing

Murine colon tissues were collected and generate single-cell gel bead in-emulsions (GEMs) using the 10× Chromium platform according to the manufacturer's protocols. Subsequent library construction and sequencing were performed. All libraries underwent paired-end sequencing with dual indexing on the NextSeq 500 platform (Illumina).

Data preprocessing

Raw scRNA-seq data were processed by Cell Ranger (v.3.1.0) software (10× Genomics) for read alignment, barcode assignment and unique molecular identifier (UMI) counting, based on genomes (mm10-3.0.0). Cells meeting criteria of UMI >200 and mitochondrial RNA <10% were retained. Subsequent analyses were performed using the Seurat package in R. Quality-controlled single-cell data were first integrated and subjected to principal component analysis (PCA) for linear dimensionality reduction. A K-nearest neighbor (KNN) graph was then constructed to refine cellular neighborhood relationships based on edge weights. Proximity distances were calculated using FindNeighbors, followed by iterative clustering with

FindClusters. Finally, the dataset was visualized through Uniform Manifold Approximation and Projection (UMAP) for non-linear dimensionality reduction. Cell clustering was conducted via FindClusters function using the top 30 principal components (PCs) to resolve cell identities. Cluster-specific marker genes were identified using the Wilcoxon rank-sum test (FindAllMarkers, default parameters) and annotated based on known markers and top-ranked genes

Pseudotime analysis

Monocle2 was applied to illustrate the developmental epithelial cells trajectories using the UMI count matrix. Cluster-specific variable genes were employed to order cells along the pseudotemporal axis. The epithelial cells cluster 3 and 8 (E03 and E08) was defined as the root state.

CellPhoneDB analysis

The analysis starts with input preparation, a single-cell expression matrix with cell-type annotations and gene symbols. Next, mean expression levels of ligands and receptors are calculated for each cell type. Finally, interaction scores are computed by multiplying the average ligand expression in a source cell type with the average receptor expression in a target cell type, yielding a quantitative measure of potential communication strength between cell populations.

Medicine and food homologous TCM screening for high-fructose exacerbated CAC

Food∩TCM is the first and most comprehensive database in China that integrates fundamental information on the medicinal sources and uses of medicine–food homologous substances with their chemical and nutritional compositions (http://101.200.131.49:8000/). Users can browse or search the database based on personal preferences, either by the name of the medicinal substance (hereafter referred to as “herb”) or by the compound name. Future versions will support additional search options, including the source of the herb as well as its descriptions and functional annotations.

The current version of Food∩TCM (v1.0) integrates data from multiple authoritative sources, including FooDB, FooDisNET, TASLY-TCM, PubChem, PubMed, the Chinese Pharmacopoeia (2020 edition), and the Chinese Food Composition Table (Standard Edition, 6th Edition). This database serves as a valuable reference and guiding resource for researchers studying medicine–food homologous substances in Traditional Chinese Medicine and for the development of related health products.

To identify targeted food with potential therapeutic effects against the key mechanisms of high-fructose exacerbated CAC, we systematically compiled data on medicine-food homologous TCM and their compound compositions from the literature and various databases. Additionally, we established an online database, Food∩TCM, to facilitate further exploration and application of these findings. In

total, 112 herbs and their related compound were documented. Before screening, the data was further processed by being annotated to PubChem database and TCM with at least one valid compound in PubChem were kept for further analysis. The compounds in these homologous were then transferred into chemical similarities between reference compounds in a network-based whole-genome drug-target prediction algorithm [17]. And the biological effect profile of every compound in each medicine-food homologous TCM was calculated by this algorithm. Further, according to our previous statistical strategy [22], the holistic targets of each medicine-food homologous TCM were obtained (adjust P value < 0.05, Benjamini-Hochberg procedure).

With the four gene sets obtained from scRNA-seq of high-fructose exacerbated CAC and predicted holistic targets of 101 medicine and food homologous, the closeness between each gene set and each medicine-food homologous TCM was estimated on the hypergeometric distribution. The candidate medicine and food homologous for high-fructose-induced CAC was screened based on the significance and mapping count. The expression pattern of Kelp in scRNA-seq was measured by the mean expression of the holistic targets of Kelp in different cell types and visualized by VlnPlot function from Seurat package in R.

Key Compound Identification from Kelp

The biological effect profiles of compounds in Kelp were kept for further analysis. In

total, 50 compounds were identified in Kelp according to our collection. And the druglikeness of these compounds were calculated by the implementation based on Python rdkit package [23]. 27 compounds with quantitative estimate of drug-likeness (QED) score no less than 0.3 were kept for further analysis. Additionally, the same as screening TCM for high-fructose exacerbated CAC, the closeness between each gene set and every compound in Kelp was also estimated on the hypergeometric distribution. The expression pattern correlations of these compounds and Kelp were estimated on the Spearman correlation based on the corresponding expression on each cell belonging to stromal cells or epithelial cells, visualized by ggplot2 package in R. The network similarity of two gene sets was implemented by GOSemsim package in R, with methods set as “Wang”.

Network target of Kelp in the treatment of high-fructose exacerbated CAC

In order to find the key mechanism of Kelp in treating high-fructose-induced CAC, network target analysis was performed based on the holistic targets of Kelp and the four gene sets. The biological network composed of certain molecules in gene set A, B, C, D, as well as the holistic targets of Kelp and CRC-related biomolecules were constructed based on protein-protein interaction data, and were then visualized by Cytoscape. KEGG enrichment was conducted to find the significantly enriched pathways in either Kelp or the four gene sets, implemented by clusterProfiler package in R. Pathways significantly enriched by the holistic targets of Kelp were then

categorized into 4 modules, including Metabolism, Environmental Information Processing, Cellular Processes and Organismal Systems. On the other hand, 10 pathways were found to be significantly enriched by the molecules in all four gene sets, and 9 of them were potentially targeted by Kelp intervention (adjust P value < 0.05, Benjamini-Hochberg procedure). The pathways were visualized using the R package ggplot2, and the -log10 of the enrichment significance (P value) was standardized for each gene set to observe more obvious differences.

The correlations of the expression of EGFR and EREG between Kelp, Fuc and dieckol were estimated on the Spearman correlation on each cell belonging to the whole detected cells, stromal cells or epithelial cells, respectively. And the expression levels of EREG and EGFR in scRNA-seq were measured and visualized by VlnPlot function from Seurat package in R, in different cell types.

H&E staining

Colon tissues were fixed in 4% paraformaldehyde for 24 hours, followed by paraffin embedding and sectioning. Paraffin sections were deparaffinized in xylene after 90-minute incubation at 65°C, then rehydrated through descending concentrations of ethanol. Sections were rinsed in distilled water before staining with hematoxylin for 1 minute. Differentiation was performed using 1% hydrochloric acid-ethanol, followed by bluing in 1% ammonia water. After rinsing, sections were counterstained with 1%

eosin and mounted with neutral balsam for microscopic examination.

Immunohistochemistry

Following deparaffinization and antigen retrieval, sections were permeabilized with 0.5% Triton X-100. Endogenous peroxidase activity was blocked, and non-specific binding was prevented using 3% goat serum. Primary antibodies were incubated at 4°C overnight, followed by species-specific secondary antibodies at room temperature for 2 hours. After PBS washes, DAB was applied, and the reaction was stopped with double-distilled water. Hematoxylin counterstaining was performed prior to mounting. All steps were optimized to ensure specificity and reproducibility.

Immunofluorescence staining

Following antigen retrieval, tissue sections or cells were permeabilized with 0.5% Triton X-100 for 1 minute, then washed with PBS on a rocking platform. After blocking non-specific binding sites, species-specific primary antibodies were applied and incubated overnight at 4°C. On the following day, sections were allowed to equilibrate to room temperature for 30 minutes. Corresponding fluorescent secondary antibodies (selected based on host species of primary antibodies) were subsequently incubated in the dark for 2 h, followed by three PBS washes. DAPI working solution (1:5000 dilution) was applied for nuclear counterstaining at room temperature for 30 minutes, then removed by washing. Finally, sections were mounted with antifade

mounting medium to preserve fluorescence signals.

Microscale thermophoresis (MST)

NIH-3T3 cells were transfected with GFP-CNBP plasmids or mutant plasmids and lysed 48 hours post-transfection. Dieckol, utilized as a ligand, was diluted twofold and combined with an equal volume of cell lysate to yield a final fluorescence intensity of 400. Binding assays were conducted using the Monolith NT.115 instrument, followed by quantitative analysis of protein-ligand interactions via MO.Affinity Analysis software.

ChIP assay

NIH-3T3 cells were transfected with GFP-CNBP plasmids and, 48 hours post-transfection, were subjected to appropriate stimulatory conditions. Following transfection, chromatin immunoprecipitation was performed. Briefly, target proteins and DNA were crosslinked using 1% formaldehyde. Cells were collected, lysed, and genomic DNA was fragmented via sonication. Reverse crosslinking was then conducted to assess the size distribution of sheared DNA fragments. After optimizing sonication conditions, chromatin immunoprecipitation was performed using Protein A+G Agarose/Salmon Sperm DNA beads. Enriched DNA fragments were subsequently quantified by qPCR. Primers were designed based on the promoter sequence of the *Ereg* gene and chromatin accessible regions identified using the

Cistrome Data Browser, ensuring targeted amplification of regulatory elements.

Cellular thermal shift assay

L929 cells were equally divided into two 10 cm dishes and cultured overnight. One dish was treated with dieckol (10 mM), while the other received 0.1% DMSO as a control. After 2 hours, cells were harvested, aliquotted into ten 200 μL PCR tubes, and subjected to heat treatment in a thermocycler. Samples were centrifuged at 13,000 × g for 20 minutes, and analyzed by Western blotting to detect target proteins.

Plasmid DNA transfection

NIH-3T3 cells were seeded into culture plates and transfected with GFP-CNBP, EREG plasmids, or corresponding vectors. All plasmids were sourced from Corues Biotechnology. Lipofectamine™ 3000 were purchased from Thermo Fisher Scientific.

ELISA assay

The protein level in mice serum was detected by an ELISA kit (ELK Biotechnology). Following initial incubation for 1.5 hours, the samples were washed, and the supernatant was discarded. The detection antibody was then added, followed by a secondary 1.5-hour incubation. After incubation with TMB substrate and stop solution, absorbance at 450 nm was measured to quantify EREG protein levels.

Cell counting kit-8 assay

Cell viability was assessed using the Cell Counting Kit-8 (CCK-8, MCE) following the manufacturer's protocol. NIH-3T3 cells were seeded in culture plates for 12 hours to transfection with the indicated EREG plasmid. After 48 hours, varying concentrations of fructose or OSMI-1 were added to the culture medium and incubated for an additional 24 hours. The supernatant was collected, centrifuged at 1000 × *g* to remove cellular debris, and then incubated with MC38 cells for 24 hours. CCK-8 solution was added to the reaction mixture, and the absorbance was measured at 450 nm using a microplate reader.

Dual Luciferase Reporter Gene Assay

NIH-3T3 cells were seeded in a 96-well plate and transfected with the indicated plasmids or vectors. After 48 hours, 100 μL of cell lysis buffer was added to each well. Following supernatant collection, 100 μL of firefly luciferase substrate was added to measure relative light units (RLU). Subsequently, 100 μL of Renilla luciferase detection reagent was added to determine RLU.

Using Renilla luciferase activity as control, the RLU value from firefly luciferase was normalized to that of Renilla luciferase. The resulting ratio was used to compare the activation levels of the reporter gene across different samples.

TRAP analysis

To identify dieckol-binding proteins in L929 cells, we implemented the Target-Responsive Accessibility Profiling (TRAP) approach. Two independent cultures of L929 cells were treated with either 50 nM TNFα alone or 10 μM dieckol combined with 50 nM TNFα. Following a 2-hour incubation period, cellular membranes were permeabilized using M-PER buffer. The resulting lysates were subjected to covalent labeling via the addition of formaldehyde and borane pyridine complex, which specifically target lysine residues in proteins under ambient conditions for accessibility profiling. Detailed methodological protocols are provided in previously published literature.

EREG degradation analysis

L929 cells were cultured in serum-free medium supplemented with 50 μg/mL cycloheximide for 1 hour. Subsequently, cells were treated with 10 mM fructose and incubated for 3, 6, or 9 hours. Following incubation, cells were lysed, and total protein extracts were subjected to Western blotting analysis to assess target protein expression levels.

Statistical analysis

The data represent the mean ± SEM, Unpaired two-tailed Student's t tests and One-

way analysis of variance (ANOVA) with Tukey multiple comparison tests were used to assess statistical significance. Statistical significance was set at $*P<0.05$, $**P<0.01$, ns, not significant.
